# Interval combined prediction of mine tunnel’s air volume considering multiple influencing factors

**DOI:** 10.1371/journal.pone.0318621

**Published:** 2025-02-07

**Authors:** Zhen Wang, Erkan Topal, Liangshan Shao, Chen Yang

**Affiliations:** 1 School of Business Administration, Liaoning Technical University, Huludao, Liaoning, China; 2 WA School of Mines: Minerals, Energy and Chemical Engineering, Curtin University, Perth, Western Australia, Australia; 3 School of Software, Liaoning Technical University, Huludao, Liaoning, China; National Institute of Technology Rourkela, INDIA

## Abstract

Continuous monitoring and accurate measurement of required air volume in mine tunnels are crucial phenomena for mine safety However, air volume fluctuates and can become unstable which can lead to biased measurement in underground environment. In this paper, to accurately measure the mine tunnel air volume, the tunnel air volume, and related ventilation parameters are consistently monitored, and the real monitoring data is converted to interval numbers for representation. These interval numbers are then preprocessed using an Interval-type Complete Ensemble Empirical Mode Decomposition with Adaptive Noise(In-CEEMDAN) to extract the essential features of the data. Then, the monitored data is processed using the phase space reconstruction technique to identify the most relevant influencing factors related to the air volume. The tunnel air volume and influencing factors are then input into different neural networks for air volume prediction. To further improve prediction accuracy, the predicted values of wind volume intervals from the single prediction method are transformed into triangular fuzzy numbers, and the generalized induced ordered weighted average operator is introduced for the combination of prediction results. The grey correlation method is selected as the optimization criterion, and the preference coefficients are used to transform the multi-objective optimization problem into a single-objective optimization problem. In order to reduce the prediction error, the L2 paradigm is combined with the gray correlation to construct a complete interval combination type air volume prediction model which considers multiple influencing factors. Finally, a sensitivity analysis was carried out to analyze the values of the preference coefficients in the model, and the final range of values was given. Experimental analysis using data from a coal mine in Inner Mongolia showed that the method could reduce Combined Weighted Mean Absolute Error(CWMAE) to a maximum of 5.0384, Combined Weighted Root of Mean Squares Error(CWRMSE) to 6.8889, and Combined Weighted Mean Absolute Percentage Error(CWMAPE) to 1.4756, which indicates that the method proposed in this study can effectively improve the prediction accuracy of the mine tunnel air volume.

## 1. Introduction

The need for a mine ventilation system increases as the depth of coal mines rises, and a subpar ventilation system intensifies heat, moisture, poisonous and explosive gasses, and dust [1, 2]. Therefore, in order to ensure safe mining operation and a healthy working environment for miners, it is imperative to continuously monitor complex and hazardous coal mine setting and accurately measure and deliver the necessary air volume to underground working environment.

Currently, a variety of sensor types have been developed to use in integrated safety monitoring and surveillance system, with several sensors used to detect mine environmental conditions [3]. Mine safety monitoring and control systems can be used to efficiently avoid and lessen the likelihood of various mishaps [4]. However, the current wired coal mine safety monitoring system has several draw backs including complex wiring, a high failure rate, susceptible to communication cable breakage, and cumbersome system maintenance [5]. These limitations of the wired system can be efficiently addressed by using wireless communication equipment in the coal mine safety monitoring and control system, which offers the benefits of flexible wiring, lower cost, and low power consumption. Furthermore, due to the harsh and complex environment of underground mine tunnels, individual sensors may malfunction, operate inaccurately, or lose sensitivity to changes in temperature, humidity, and dust. In these instances, the system’s inability to respond to the environmental changes could lead to poor decision making in coal mine management system.

The most common industries using data mining for research and decision-making are marketing [6], banking [7], and healthcare [8] with a steady increase in recent years. However, data mining techniques are still in their infancy in the industrial and energy sectors. Numerous wind prediction strategies are being researched to increase the accuracy of the wind speed prediction. However, because of the influence from the environment and the sensors’ sensitivity, the initial wind speed series transmitted by the sensors is noisy and unstable. As a result, using the original wind speed series to construct the prediction model increases the forecast error. The primary characteristics of the original wind speed series must be examined and considered before developing an accurate forecasting model. One technique for adaptive signal separation is Empirical Modal Decomposition (EMD), which makes it possible to break down smooth signals into many intrinsic modal functions (IMF) whose instantaneous frequencies are essential and a residual trend. EMD adaptively extracts IMFs from high to low frequencies and has been widely used in many fields. However, it suffers from model aliasing. To solve this problem, Ensemble Empirical Mode Decomposition (EEMD) [9] can naturally separate scales without requiring any a priori criterion selection. This is achieved by filtering the set of signals with added white noise. The average value obtained from this process effectively leverages the statistical features of white noise. By incorporating uniform phase sinusoids as hidden signals to lessen the mode splitting impact, the uniform phase empirical mode decomposition not only overcomes the significant mixing problem present in EMD but also achieves lower computational complexity and lowers or eliminates residual noise [10].

Methods based on decomposition theory can inhibit the influence of random volatility of the tunnel wind volume on the prediction to a certain extent [11, 12]. However, it is generally only for a single tunnel wind volume data, and the improvement effect depends on the goodness of the decomposition method. Tunnel air volume source data is usually historical data from a single sensor, while the change in tunnel air volume is greatly affected by the environment and has characteristics of non-linearity and time-variability. It is crucial to address the problem of effectively extracting the correlation characteristics among the factors affecting the trend of air volume change in the tunnel and establishing an optimal prediction model to improve its accuracy and robustness. The multifactorial change of the tunnel wind volume seems to be irregular, but they present chaotic solid characteristics. With the help of the phase space reconstruction (PSR) method [13, 14] in the chaos theory, we can extract the characteristics of the chaotic time series that change with time, and improve the prediction performance of the original model [15]. For time series with multiple correlated features, multivariate phase space reconstruction can further exploit the coupling properties of multiple correlated features in time [16]. The prediction process is basically the same in different field: first, preprocessing the data; second, performing phase space reconstruction of multivariate, then constructing the prediction model by using intelligent algorithms; and finally, error analysis.

The application of intelligent algorithms also plays a crucial role in the accuracy of the prediction. Artificial intelligence and machine learning methods are frequently used to predict air wind speed and/or the volume [17–19]. These techniques offer greater flexibility in prediction applications and are better equipped with handling nonlinear relationships between inputs and outputs. However, these methods describe relationships between values implicitly, which can occasionally lead to significant computations. None of the prediction method outperforms better than others in every instance. Each prediction method has its own pros and cons which makes it suitable for specific cases. Consequently, the air volume of in tunnels is predicted using the combined prediction approach, which completely utilizes the valuable information of each prediction model thereby enhance the prediction accuracy. In combinatorial prediction theory, numerous techniques for information aggregation have been proposed [20]. Yager [21] initially proposed the ordered weighted average (OWA) operator, which offers several operators for parameter aggregation. Since its introduction, the OWA operator has been extensively researched and used in different applications [21–25].

In this research, we first gather multi-factor sensor data related to the wind volume in a mine tunnel. Then, we use interval numbers to represent the data values measured by multiple sensors over a given period of time, which eliminates the influence of inconsistency between sensor monitoring and uploading time. We then embed the interval number theory into CEEMDAN(In-CEEMDAN), enabling In-CEEMDAN to decompose the data and extract the internal features from the interval sequences. Next, we analyze the data correlation coefficients to remove the influence of excessive factors on air volume prediction and identify which correlation factors are strongly and moderately correlated. The resulting factors are then reconstructed in phase space, and the reconstructed data are used as inputs for a single prediction using four prediction methods namely CNN, LSTM, GRU, and SNN. This process eliminates the influence of too many factors on the air volume forecasting. By merging the predicted values from the four intelligence algorithms and utilizing the gray correlation degree as the optimization criterion, a multi-objective planning model is developed for the left, center, and proper ends of the triangle fuzzy number. A two-dimensional generalized induced ordered weighted logarithmic average operator is then developed. Then, the the multi-objective planning model is converted into a single-objective planning model using the preference coefficient which accounts for the ambiguity and uncertainty in the actual and anticipated mine tunnel air volume data. Furthermore, the L2 paradigm is combined with the interval prediction model’s gray correlation degree to prevent the "enlargement" or "shrinkage" effect of prediction errors and the scenario where the left endpoint of the interval is larger than the right endpoint following the combined prediction. This results in the construction of a comprehensive combined interval prediction model for the mine tunnel’s air volume. Three sets of comparative tests demonstrate the efficiency of the combined prediction approach proposed in this research.

## 2. Empirical model decomposition of complete systems with adaptive noise based on the number of intervals

Although Empirical Mode Decomposition(EMD) [26] technique can decompose nonlinear data into modal components with distinct frequencies, it often struggles to split signals in real-world scenarios and contains mode confusion issues. In order to address these issues, Torres [27] proposed the CEEMDAN algorithm, which superimposes pairs of white noises containing the control parameter *ξ*_*i*_ onto the original signal. This algorithm not only solves the modal confusion issue in EMD, but it also operates at a faster computational speed than EEMD. In addition to reconstructing the noise-free preference and resolving the mode confusion issue of EMD, the equalization property computes more quickly than EEMD.

Due to the influence of sensor uploading times, it is not possible to collect data from every sensor at the same time. In order to overcome to this obstacle, we use the interval number rather than the real number. The upper and lower limits of these interval number are set to the maximum and minimum values of the data obtained in a given time interval.

Definition 1: Let $a=[a^{-},a^{+}],\{x|a^{-}\leq x\leq a^{+};a^{-},a^{+}\in R\}$ and call *a* an interval number [28]. Let *a* = [*a*^−^,*a*^+^], *b* = [*b*^−^,*b*^+^], then:

$$a+b=[a^{-}+b^{-},a^{+}+b^{+}]$$ (1)

$$a-b=[a^{-}-b^{+},a^{+}-b^{-}]$$ (2)

$$\lambda a=[\lambda a^{-},\lambda a^{+}]$$ (3)

The interval number is embedded within it in this work, which is based on CEEEMDAN to get the In-CEEMDAN approach as follows:

Step 1: $X(t)=[x^{-}(t),x^{+}(t)]$ is the original data signal, and Gaussian white noise *w*_*i*_(*t*) with amplitude (standard deviation) *ε*_0_ is added to the original signal. *w*_*i*_(*t*), *i* = 1,2,⋯,*I* is the set of Gaussian white noise sequences with zero mean and unit variance. That is:

$$X{\left(t\right)}^{\prime }=X(t)+\varepsilon_{0}w_{i}(t)\to \{\begin{array}{c} x^{-}(t{)}^{\prime }=x^{-}(t)+\varepsilon_{0}w_{i}(t)\\ x^{+}(t{)}^{\prime }=x^{+}(t)+\varepsilon_{0}w_{i}(t) \end{array}$$ (4)

Step 2: Use a new operator *E*_*j*_(***X***) for the *j*-th eigenmode component obtained after EMD decomposition.

Step 3: The first mode of In-CEEMDAN is computed by performing *i*-th (number of trials) EMD decompositions of $X(t{)}^{\prime }=[x^{-}(t{)}^{\prime },x^{+}(t{)}^{\prime }]$, the results of which are averaged to obtain the first intrinsic modal component, $IMF_{1}(t)=[imf_{1}^{-}(t),imf_{1}^{+}(t)]$, with the following equation:

$$E_{1}[X(t{)}^{\prime }]=IMF_{1}^{i}(t)\to \{\begin{array}{c} E_{1}[x^{-}(t{)}^{\prime }]=imf_{1i}^{-}(t)\\ E_{1}[x^{+}(t{)}^{\prime }]=imf_{1i}^{+}(t) \end{array}$$ (5)

$$IMF_{1}(t)=\frac{1}{I}\sum_{i=1}^{I}IMF_{1}^{i}(t)\to \{\begin{array}{c} imf_{1}^{-}(t)=\frac{1}{I}\sum_{i=1}^{I}imf_{1i}^{-}(t)\\ imf_{1}^{+}(t)=\frac{1}{I}\sum_{i=1}^{I}imf_{1i}^{+}(t) \end{array}$$ (6)

Step 4: The first residual can be obtained by calculating the removal of the first modal $IMF_{1}(t)=[imf_{1}^{-}(t),imf_{1}^{+}(t)]$:

$$R_{1}(t)=X(t{)}^{\prime }-IMF_{1}(t)\to \{\begin{array}{c} r_{1}^{-}(t)=x^{-}(t{)}^{\prime }-imf_{1}^{-}(t)\\ r_{1}^{+}(t)=x^{+}(t{)}^{\prime }-imf_{1}^{+}(t) \end{array}$$ (7)

Step 5: Decomposition strategy to get $R_{1}(n)+\varepsilon_{1}E_{1}(w_{i}(n)),i=1,2,\cdots ,I$, the second eigenmode component $IMF_{2}(t)=[imf_{2}^{-}(t),imf_{2}^{+}(t)]$ and residuals are calculated, i.e:

$$IMF_{2}(t)=\frac{1}{I}\sum_{i=1}^{I}E_{1}(R_{1}(t)+\varepsilon_{1}E_{1}(w_{i}(t)))\to \{\begin{array}{c} imf_{2}^{-}(t)=\frac{1}{I}\sum_{i=1}^{I}E_{1}(r_{1}^{-}(t)+\varepsilon_{1}E_{1}(w_{i}(t)))\\ imf_{2}^{+}(t)=\frac{1}{I}\sum_{i=1}^{I}E_{1}(r_{1}^{+}(t)+\varepsilon_{1}E_{1}(w_{i}(t))) \end{array}$$ (8)

$$R_{2}(t)=R_{1}(t)-IMF_{2}(t)\to \{\begin{array}{c} r_{2}^{-}(t)=r_{1}^{-}(t)-imf_{2}^{-}(t)\\ r_{2}^{+}(t)=r_{1}^{+}(t)-imf_{2}^{+}(t) \end{array}$$ (9)

Step 6: For the other stages, the above steps are repeated to calculate the *k*-th eigenmode component and the residual value, which is realized with the following equation:

$$IMF_{k}(t)=\frac{1}{I}\sum_{i=1}^{I}E_{1}(R_{k-1}(t)+\varepsilon_{k-1}E_{k-1}(w_{i}(t)))\to \{\begin{array}{c} imf_{k}^{-}(t)=\frac{1}{I}\sum_{i=1}^{I}E_{1}(r_{k-1}^{-}(t)+\varepsilon_{k-1}E_{k-1}(w_{i}(t)))\\ imf_{k}^{+}(t)=\frac{1}{I}\sum_{i=1}^{I}E_{1}(r_{k-1}^{+}(t)+\varepsilon_{k-1}E_{k-1}(w_{i}(t))) \end{array}$$ (10)

$$R_{k}(t)=R_{k-1}(t)-IMF_{k}(t)\to \{\begin{array}{c} r_{k}^{-}(t)=r_{k-1}^{-}(t)-imf_{k}^{-}(t),k=2,3,\cdots ,K\\ r_{k}^{+}(t)=r_{k-1}^{+}(t)-imf_{k}^{+}(t),k=2,3,\cdots ,K \end{array}$$ (11)

Step 7: The counting process of *K*+1 modes is represented by the following equation:

$$IMF_{k+1}(t)=\frac{1}{I}\sum_{i=1}^{I}E_{1}(R_{k}(t)+\varepsilon_{k}E_{k}(w_{i}(t)))\to \{\begin{array}{c} imf_{k+1}^{-}(t)=\frac{1}{I}\sum_{i=1}^{I}E_{1}(r_{k}^{-}(t)+\varepsilon_{k}E_{k}(w_{i}(t)))\\ imf_{k+1}^{+}(t)=\frac{1}{I}\sum_{i=1}^{I}E_{1}(r_{k}^{+}(t)+\varepsilon_{k}E_{k}(w_{i}(t))) \end{array}$$ (12)

Step 8: Loop through steps 6–8, stopping the step when the residuals become a monotonic function, as extraction of the ***IMF*** components cannot be performed at this point. The original signal $X(t)=[x^{-}(t),x^{+}(t)]$ can be reconstructed as:

$$X(t)=\sum_{k=1}^{K}IMF_{k}+R_{k}(t)\to \{\begin{array}{c} x^{-}(t)=\sum_{k=1}^{K}imf_{k}^{-}+r_{k}^{-}(t)\\ x^{+}(t)=\sum_{k=1}^{K}imf_{k}^{+}+r_{k}^{+}(t) \end{array}$$ (13)

In-CEEMDAN extracts the intrinsic characteristics of the data by using the steps above to process raw data from various sensor types.

## 3. Multi-sensor variable phase space reconstruction

The inherent law of chaotic time series, which can transform a time series with chaotic properties into a nonlinear time series with higher dimensions, are often studied through phase space reconstruction [29]. In real case applications, the univariate phase space reconstruction of the tunnel air volume cannot adequately capture its chaotic properties due to chaotic features of the time series, resulting in a significant prediction error. The meteorological factors affecting the tunnel air volume should be considered and integrated into the multivariate time series with the tunnel air volume in order to realize the multivariate phase space reconstruction. Furthermore, this approach will restore the nonlinear chaotic system of the tunnel air volume, and improve the prediction accuracy [30]. In order to perform multivariate spatial reconstruction and obtain the direct multivariate correlation, the interval number can be represented by its midpoint i.e., $x=\left(x^{-}+x^{+}\right)/2$.

Together, the tunnel air volume time series *Y*_1_ and the *n* influencing factors *Y*_2_,*Y*_3_,⋯,*Y*_*n*+1_ form a *D*-dimensional multivariate time series {*Y*_*i*_,*i* = 1,2,⋯,*D*}, where $Y_{i}=[y_{i}(1),y_{i}(2),\cdots ,y_{i}(N)]$ and *N* is the time series length. Embedding dimension *m*_*i*_ and delay time *τ*_*i*_ of the univariate time series are then calculated, respectively, and the *D*-dimensional multivariate time series’ phase space is established as follows:

$$V_{n}=[\begin{array}{l} y_{1}(n),y_{1}(n-\tau_{1}),\cdots ,y_{1}(n-(m_{1}-1)\tau_{1}),\\ y_{2}(n),y_{2}(n-\tau_{2}),\cdots ,y_{2}(n-(m_{2}-1)\tau_{2}),\\ \cdots \\ y_{D}(n),y_{D}(n-\tau_{D}),\cdots ,y_{D}(n-(m_{D}-1)\tau_{D}), \end{array}]$$ (14)

Where *m*_*i*_ and *τ*_*i*_ are the embedding dimension and delay time of the *i*-th time series, respectively; $n=\underset{1\leq i\leq D}{\max}(m_{i}-1)\tau_{i}+1$, the dimension of the phase space after reconstruction, $d=\sum_{i=1}^{D}m_{i}$.

To better restore the inner evolution law of the nonlinear chaotic model of the tunnel air volume, each phase point of the reconstructed multivariate phase space contains more diverse information about the tunnel air volume than the univariate tunnel air volume time series. This is because each phase point of the multivariate phase space integrates the influence factors related to the air volume into this type of process.

Determining the delay time and embedding dimension of each feature time series of the air volume is crucial to reconstructing the multivariate phase space of the air volume prediction time series. An optimization approach is required to estimate the delay time and embedding dimension of the time series in order to correctly portray the temporal coupling connection between the tunnel air volume and meteorological parameters. The C-C method is one of the most popular and accurate methods for calculating delay times. It uses the time series’ correlation integral to create a nonlinear time series correlation statistic from which the embedding window *τ*_*W*_ and delay time *τ* can be calculated. Then the embedding dimension *m* can be obtained by the embedding window method [31, 32].

## 4. Modeling of air volume combination prediction

To forecast the tunnel’s air volume, we employed three widely used neural network techniques in this research: CNN [33], LSTM [34], GRU [35], and SNN [36]. These techniques were integrated with data preprocessing and phase space reconstruction to obtain four prediction approaches. The limitation of using actual numbers as the forecast results can be addressed by using three distinct prediction techniques which will provide three sets of air volume interval values [37]. However, relaying on the single prediction approach can be risky, as poor selection can lead to a more significant final prediction error and more impact on our estimation. This shortcoming can be mitigated by a combination prediction approach, which generally yields more accurate prediction outcomes than the single prediction method.

Definition 2: We get $U=\{\langle u_{1},a_{1}\rangle ,\langle u_{2},a_{2}\rangle ,\cdots ,\langle u_{m},a_{m}\rangle \}$, the induced two-dimensional array. When $f_{w}(\langle u_{1},a_{1}\rangle ,\langle u_{2},a_{2}\rangle ,\cdots ,\langle u_{m},a_{m}\rangle )=\exp{\left(\sum_{i=1}^{m}w_{i}{\left(\ln b_{i}\right)}^{\lambda }\right)}^{1/\lambda }$ holds, the function *f*_*w*_ is referred to as a two-dimensional GIOWLA operator, which is a generalized induced ordered weighted logarithmic average operator. In decreasing sequence based on the magnitude of *u*_*i*_, *b*_*i*_ is the *i*-th value of *a*_1_,*a*_2_,⋯,*a*_*m*_, and *u*_*i*_ is the induced value of *a*_*i*_. Since *w*_*i*_ is exclusively connected to the position of the induced value, the weighted vector *W* = (*w*_1_,*w*_2_,⋯,*w*_*m*_) satisfies $\sum_{i=1}^{m}w_{i}=1,w_{i}\leq 0$ and λ∈(−∞,0)∪(0,+∞).

The actual interval sequence of air volume is $\left\{Q_{t}=(q_{t}^{-},q_{t}^{+})|t=1,2,\cdots ,n\right\}$, which is predicted by *m* single forecasting methods. Let *Q*_*it*_ be the prediction sequence value of the *i*-th prediction method at moment *t*, then $\left\{Q_{it}=(q_{it}^{-},q_{it}^{+})|i=1,2,\cdots ,m;t=1,2,\cdots ,n\right\}$. If ${\hat{Q}}_{t}=\sum_{i=1}^{m}w_{i}Q_{it}$, $\left\{{\hat{Q}}_{t}=({\hat{q}}_{t}^{-},{\hat{q}}_{t}^{+})|t=1,2,\cdots ,n\right\}$ is the combined prediction value of the air volume sequence, where *w*_1_,*w*_2_,⋯,*w*_*m*_ is the weight coefficient of each single prediction in the combined prediction, and $\sum_{i=1}^{m}w_{i}=1,w_{i}\leq 0$ is satisfied.

The air volume in mine tunnel has got a characteristic of ambiguity, uncertainty, and unpredictability. Therefore, it can be assumed that there is an equal probability of finding the air volume value at any point within the interval. Consequently, an interval-based combined air volume prediction model can be constructed by converting the air volume’s threshold interval into a triangular fuzzy number. For the purpose of air volume prediction, the triangular fuzzy numbers $\left\{{\tilde{Q}}_{t}=(q_{t}^{-},q_{t},q_{t}^{+})|t=1,2,\cdots ,n\right\}$ and $q_{t}=\left(q_{t}^{-}+q_{t}^{+}\right)/2$ match the actual sequence of intervals. The air volume sequence for the *i*-th air volume prediction method at moment *t* by triangular fuzzy number is $\left\{{\tilde{Q}}_{it}=(q_{it}^{-},q_{it},q_{it}^{+})|i=1,2,\cdots ,m;t=1,2,\cdots ,n\right\}$, and the corresponding combination air volume prediction sequence at time *t* by triangular fuzzy number is $\left\{{\hat{Q}}_{t}=({\hat{q}}_{t}^{-},{\hat{q}}_{t},{\hat{q}}_{t}^{+})|t=1,2,\cdots ,n\right\}$.

Definition 3: Let $u_{it}^{-}$, *u*_*it*_, and $u_{it}^{+}$ be the left endpoint accuracy, middle point accuracy and right endpoint accuracy of the single prediction triangle fuzzy number of the *i*-th prediction method at the moment *t*,

$$u_{it}^{-}=\{\begin{array}{cc} 1-|\frac{\left(q_{t}^{-}-q_{it}^{-}\right)}{q_{t}^{-}}| & |\frac{\left(q_{t}^{-}-q_{it}^{-}\right)}{q_{t}^{-}}|<1\\ 0 & |\frac{\left(q_{t}^{-}-q_{it}^{-}\right)}{q_{t}^{-}}|\geq 1 \end{array}\;\begin{array}{c} i=1,2,\cdots ,m\\ n=1,2,\cdots ,n \end{array}$$ (15)

$$u_{it}=\{\begin{array}{cc} 1-|\frac{\left(q_{t}-q_{it}\right)}{q_{t}}| & |\frac{\left(q_{t}-q_{it}\right)}{q_{t}^{-}}|<1\\ 0 & |\frac{\left(q_{t}-q_{it}\right)}{q_{t}}|\geq 1 \end{array}\;\begin{array}{c} i=1,2,\cdots ,m\\ t=1,2,\cdots ,n \end{array}$$ (16)

$$u_{it}^{+}=\{\begin{array}{cc} 1-|\frac{\left(q_{t}^{+}-q_{it}^{+}\right)}{q_{t}^{-}}| & |\frac{\left(q_{t}^{+}-q_{it}^{+}\right)}{q_{t}^{+}}|<1\\ 0 & |\frac{\left(q_{t}^{+}-q_{it}^{+}\right)}{q_{t}^{+}}|\geq 1 \end{array}\;\begin{array}{c} i=1,2,\cdots ,m\\ t=1,2,\cdots ,n \end{array}$$ (17)

According to Eqs (15), (16) and (17), it can be seen that the values of $u_{it}^{-}$, *u*_*it*_, and $u_{it}^{+}$ are in the range from 0 to 1. If $u_{it}^{-}$ is regarded as the inducement value of $q_{it}^{-}$, then the prediction accuracy of *m* single prediction methods at moment *t* and its corresponding dimensionless data constitute *m* two-dimensional arrays $\left\{\langle u_{1t}^{-},q_{1t}^{-}\rangle ,\langle u_{2t}^{-},q_{2t}^{-}\rangle ,\cdots ,\langle u_{mt}^{-},q_{mt}^{-}\rangle \right\}$. According to definition 2, there are:

q^t−=fw{〈u1t−,q1t−〉,〈u2t−,q2t−〉,⋯,〈umt−,qmt−〉}=exp{(∑i=1mwi(lnbit−)λ)1λ} (18)

${\hat{q}}_{t}^{-}$ is said to be the GIOWLA combined prediction value of the left end point of the air volume interval generated by the precision sequence $u_{1t}^{-},u_{2t}^{-},\cdots ,u_{mt}^{-}$ as the induction variable, where $b_{it}^{-}$ is the *i* largest prediction accuracy value among $u_{1t}^{-},u_{2t}^{-},\cdots ,u_{mt}^{-}$.

The same can be said for:

q^t=fw{〈u1t,q1t〉,〈u2t,q2t〉,⋯,〈umt,qmt〉}=exp{(∑i=1mwi(lnbit)λ)1λ} (19)

q^t+=fw{〈u1t+,q1t+〉,〈u2t+,q2t+〉,⋯,〈umt+,qmt+〉}=exp{(∑i=1mwi(lnbit+)λ)1λ} (20)

*b*_*it*_ and $b_{it}^{+}$ are the *i*-th number arranged in descending order according to the magnitude of induction precision *u*_1*t*_,*u*_2*t*_,⋯,*u*_*mt*_ and $u_{1t}^{+},u_{2t}^{+},\cdots ,u_{mt}^{+}$, respectively. *W* = (*w*_1_,*w*_2_,⋯,*w*_*m*_) is the weighting vector of the GIOWLA operator and satisfies $\sum_{i=1}^{m}w_{i}=1,w_{i}\leq 0$.

Definition 4: $e_{it}={\left(\ln\;q_{t}\right)}^{\lambda }-{\left(\ln\;{\hat{q}}_{it}\right)}^{\lambda }$ is the prediction error of the logarithmic *λ*-power of the *i* single prediction method at moment *t*. $e_{t}^{-}={\left(\ln q_{t}^{-}\right)}^{\lambda }-{\left(\ln{\hat{q}}_{t}^{-}\right)}^{\lambda }$, $e_{t}={\left(\ln q_{t}\right)}^{\lambda }-{\left(\ln{\hat{q}}_{t}\right)}^{\lambda }$, and $e_{t}^{+}={\left(\ln q_{t}^{+}\right)}^{\lambda }-{\left(\ln{\hat{q}}_{t}^{+}\right)}^{\lambda }$ are respectively the prediction errors of the logarithmic *λ*-power of the left, middle and right endpoints of GIOWLA’s combined forecast of air volume at the moment *t*.

The L2 paradigm can be combined with the gray correlation of the interval prediction model, the L2 paradigm expression for the ${\left\|X\right\|}_{2}=\sqrt{\sum_{i=1}^{n}x_{i}^{2}}$, to avoid the "enlargement" or "shrinkage" effect of the prediction error and the emergence of the predicted number of intervals of the left endpoint is greater than the right endpoint of the situation. Subsequently, the single prediction method based on the L2 paradigm predicts the left, mid, and right endpoint prediction errors, respectively:

$$\begin{array}{l} {\|{e_{t}}^{-}\|}_{2}=\sqrt{\sum_{t=1}^{n}{\left({\left(\ln\;q_{t}^{-}\right)}^{\lambda }-{\left(\ln\;{\hat{q}}_{t}^{-}\right)}^{\lambda }\right)}^{2}}=\sqrt{\sum_{t=1}^{n}{\left({\left(\ln\;q_{t}^{-}\right)}^{\lambda }-\sum_{i=1}^{m}w_{i}{\left(\ln\;q_{it}^{-}\right)}^{\lambda }\right)}^{2}}\\ \;=\sqrt{\sum_{t=1}^{n}{\left(\sum_{i=1}^{m}w_{i}({\left(\ln\;q_{t}^{-}\right)}^{\lambda }-{\left(\ln\;q_{it}^{-}\right)}^{\lambda })\right)}^{2}}=\sqrt{\sum_{i=1}^{m}\sum_{j=1}^{m}w_{i}w_{j}(\sum_{t=1}^{n}\left(\left({\left(\ln\;q_{t}^{-}\right)}^{\lambda }-{\left(\ln\;q_{it}^{-}\right)}^{\lambda })({\left(\ln\;q_{t}^{-}\right)}^{\lambda }-{\left(\ln\;q_{jt}^{-}\right)}^{\lambda }\right)\right))} \end{array}$$ (21)

$$\begin{array}{l} {\left\|e_{t}\right\|}_{2}=\sqrt{\sum_{t=1}^{n}{\left({\left(\ln\;q_{t}\right)}^{\lambda }-{\left(\ln\;{\hat{q}}_{t}\right)}^{\lambda }\right)}^{2}}=\sqrt{\sum_{t=1}^{n}{\left({\left(\ln\;q_{t}\right)}^{\lambda }-\sum_{i=1}^{m}w_{i}{\left(\ln\;q_{it}\right)}^{\lambda }\right)}^{2}}\\ \;=\sqrt{\sum_{t=1}^{n}{\left(\sum_{i=1}^{m}w_{i}({\left(\ln\;q_{t}\right)}^{\lambda }-{\left(\ln\;q_{it}\right)}^{\lambda })\right)}^{2}}=\sqrt{\sum_{i=1}^{m}\sum_{j=1}^{m}w_{i}w_{j}(\sum_{t=1}^{n}\left(\left({\left(\ln\;q_{t}\right)}^{\lambda }-{\left(\ln\;q_{it}\right)}^{\lambda })({\left(\ln\;q_{t}\right)}^{\lambda }-{\left(\ln\;q_{it}\right)}^{\lambda }\right)\right))} \end{array}$$ (22)

$$\begin{array}{l} {\|{e_{t}}^{+}\|}_{2}=\sqrt{\sum_{t=1}^{n}{\left({\left(\ln\;q_{t}^{+}\right)}^{\lambda }-{\left(\ln\;{\hat{q}}_{t}^{+}\right)}^{\lambda }\right)}^{2}}=\sqrt{\sum_{t=1}^{n}{\left({\left(\ln\;q_{t}^{+}\right)}^{\lambda }-\sum_{i=1}^{m}w_{i}{\left(\ln\;q_{it}^{+}\right)}^{\lambda }\right)}^{2}}\\ \;=\sqrt{\sum_{t=1}^{n}{\left(\sum_{i=1}^{m}w_{i}({\left(\ln\;q_{t}^{+}\right)}^{\lambda }-{\left(\ln\;q_{it}^{+}\right)}^{\lambda })\right)}^{2}}=\sqrt{\sum_{i=1}^{m}\sum_{j=1}^{m}w_{i}w_{j}(\sum_{t=1}^{n}\left(\left({\left(\ln\;q_{t}^{+}\right)}^{\lambda }-{\left(\ln\;q_{it}^{+}\right)}^{\lambda })({\left(\ln\;q_{t}^{+}\right)}^{\lambda }-{\left(\ln\;q_{it}^{+}\right)}^{\lambda }\right)\right))} \end{array}$$ (23)

Definition 5: Let

$$\gamma =\frac{1}{n-1}\sum_{t=2}^{n}\frac{1}{1+\varphi |e_{t}|+\rho |e_{t}-e_{t-1}|}=\frac{1}{n-1}\sum_{t=2}^{n}\frac{1}{1+\varphi \left|{\left(\ln\;q_{t}\right)}^{\lambda }-{\left(\ln\;{\hat{q}}_{t}\right)}^{\lambda }\right|+\rho |{\left(\ln\;q_{t-1}\right)}^{\lambda }-{\left(\ln\;{\hat{q}}_{t-1}\right)}^{\lambda }|}$$ (24)

We designate *γ* as the gray correlation between the combined prediction method’s actual sequence *q*_*t*_ and the series of forecasted values ${\hat{q}}_{t}$. Resolved values *φ* and *ρ* are often assumed to be 0.5 for *φ* and 1 for *ρ*. The combined prediction method’s accuracy increases with *γ*. Without a doubt, *γ* depends on the weighting vector *W*.

Using the combined prediction approach, the gray correlation between left, mid, and right endpoints of the air volume interval is stated as follows:

$$\begin{array}{l} \gamma^{-}=\frac{1}{n-1}\sum_{t=2}^{n}\frac{1}{1+\varphi {\left\|e_{t}^{-}\right\|}_{2}+\rho {\left\|e_{t}^{-}-e_{t-1}^{-}\right\|}_{2}}=\frac{1}{n-1}\sum_{t=2}^{n}\frac{1}{1+\varphi \sqrt{{\left({\left(\ln\;q_{t}^{-}\right)}^{\lambda }-{\left(\ln\;{\hat{q}}_{t}^{-}\right)}^{\lambda }\right)}^{2}}+\rho \sqrt{{\left(\left({\left(\ln\;q_{t}^{-}\right)}^{\lambda }-{\left(\ln\;{\hat{q}}_{t}^{-}\right)}^{\lambda })-({\left(\ln\;q_{t-1}^{-}\right)}^{\lambda }-{\left(\ln\;{\hat{q}}_{t-1}^{-}\right)}^{\lambda }\right)\right)}^{2}}}\\ \;=\frac{1}{n-1}\sum_{t=2}^{n}\frac{1}{1+\varphi \sqrt{{\left(\sum_{i=1}^{m}w_{i}({\left(\ln\;q_{t}^{-}\right)}^{\lambda }-{\left(\ln\;q_{it}^{-}\right)}^{\lambda })\right)}^{2}}+\rho \sqrt{{\left(\left(\sum_{i=1}^{m}w_{i}({\left(\ln\;q_{t}^{-}\right)}^{\lambda }-{\left(\ln\;q_{it}^{-}\right)}^{\lambda }))-(\sum_{i=1}^{m}w_{i}({\left(\ln\;q_{t-1}^{-}\right)}^{\lambda }-{\left(\ln\;q_{it-1}^{-}\right)}^{\lambda })\right)\right)}^{2}}}\\ \;=\frac{1}{n-1}\sum_{t=2}^{n}\frac{1}{\left(\begin{array}{l} 1+\varphi \sqrt{\sum_{i=1}^{m}\sum_{j=1}^{m}w_{i}w_{j}(\left({\left(\ln\;q_{t}^{-}\right)}^{\lambda }-{\left(\ln\;q_{it}^{-}\right)}^{\lambda })({\left(\ln\;q_{t}^{-}\right)}^{\lambda }-{\left(\ln\;q_{jt}^{-}\right)}^{\lambda }\right))}\\ +\rho \sqrt{\begin{array}{l} \sum_{i=1}^{m}\sum_{j=1}^{m}w_{i}w_{j}(\left({\left(\ln\;q_{t}^{-}\right)}^{\lambda }-{\left(\ln\;q_{it}^{-}\right)}^{\lambda })({\left(\ln\;q_{t}^{-}\right)}^{\lambda }-{\left(\ln\;q_{jt}^{-}\right)}^{\lambda }\right))\\ -\sum_{i=1}^{m}\sum_{j=1}^{m}w_{i}w_{j}(\left({\left(\ln\;q_{t-1}^{-}\right)}^{\lambda }-{\left(\ln\;q_{it-1}^{-}\right)}^{\lambda })({\left(\ln\;q_{t-1}^{-}\right)}^{\lambda }-{\left(\ln\;q_{jt-1}^{-}\right)}^{\lambda }\right)) \end{array}} \end{array}\right)} \end{array}$$ (25)

$$\begin{array}{l} \gamma =\frac{1}{n-1}\sum_{t=2}^{n}\frac{1}{1+\varphi {\left\|e_{t}\right\|}_{2}+\rho {\left\|e_{t}-e_{t-1}\right\|}_{2}}=\frac{1}{n-1}\sum_{t=2}^{n}\frac{1}{1+\varphi \sqrt{{\left({\left(\ln\;q_{t}\right)}^{\lambda }-{\left(\ln\;{\hat{q}}_{t}\right)}^{\lambda }\right)}^{2}}+\rho \sqrt{{\left(\left({\left(\ln\;q_{t}\right)}^{\lambda }-{\left(\ln\;{\hat{q}}_{t}\right)}^{\lambda })-({\left(\ln\;q_{t-1}\right)}^{\lambda }-{\left(\ln\;{\hat{q}}_{t-1}\right)}^{\lambda }\right)\right)}^{2}}}\\ \;=\frac{1}{n-1}\sum_{t=2}^{n}\frac{1}{1+\varphi \sqrt{{\left(\sum_{i=1}^{m}w_{i}({\left(\ln\;q_{t}\right)}^{\lambda }-{\left(\ln\;q_{it}\right)}^{\lambda })\right)}^{2}}+\rho \sqrt{{\left(\left(\sum_{i=1}^{m}w_{i}({\left(\ln\;q_{t}\right)}^{\lambda }-{\left(\ln\;q_{it}\right)}^{\lambda }))-(\sum_{i=1}^{m}w_{i}({\left(\ln\;q_{t-1}\right)}^{\lambda }-{\left(\ln\;q_{it-1}\right)}^{\lambda })\right)\right)}^{2}}}\\ \;=\frac{1}{n-1}\sum_{t=2}^{n}\frac{1}{\left(\begin{array}{l} 1+\varphi \sqrt{\sum_{i=1}^{m}\sum_{j=1}^{m}w_{i}w_{j}(\left({\left(\ln\;q_{t}\right)}^{\lambda }-{\left(\ln\;q_{it}\right)}^{\lambda })({\left(\ln\;q_{t}\right)}^{\lambda }-{\left(\ln\;q_{jt}\right)}^{\lambda }\right))}\\ +\rho \sqrt{\begin{array}{l} \sum_{i=1}^{m}\sum_{j=1}^{m}w_{i}w_{j}(\left({\left(\ln\;q_{t}\right)}^{\lambda }-{\left(\ln\;q_{it}\right)}^{\lambda })({\left(\ln\;q_{t}\right)}^{\lambda }-{\left(\ln\;q_{jt}\right)}^{\lambda }\right))\\ -\sum_{i=1}^{m}\sum_{j=1}^{m}w_{i}w_{j}(\left({\left(\ln\;q_{t-1}\right)}^{\lambda }-{\left(\ln\;q_{it-1}\right)}^{\lambda })({\left(\ln\;q_{t-1}\right)}^{\lambda }-{\left(\ln\;q_{jt-1}\right)}^{\lambda }\right)) \end{array}} \end{array}\right)} \end{array}$$ (26)

$$\begin{array}{l} \gamma^{+}=\frac{1}{n-1}\sum_{t=2}^{n}\frac{1}{1+\varphi {\left\|e_{t}^{+}\right\|}_{2}+\rho {\left\|e_{t}^{+}-e_{t-1}^{+}\right\|}_{2}}=\frac{1}{n-1}\sum_{t=2}^{n}\frac{1}{1+\varphi \sqrt{{\left({\left(\ln\;q_{t}^{+}\right)}^{\lambda }-{\left(\ln\;{\hat{q}}_{t}^{+}\right)}^{\lambda }\right)}^{2}}+\rho \sqrt{{\left(\left({\left(\ln\;q_{t}^{+}\right)}^{\lambda }-{\left(\ln\;{\hat{q}}_{t}^{+}\right)}^{\lambda })-({\left(\ln\;q_{t-1}^{+}\right)}^{\lambda }-{\left(\ln\;{\hat{q}}_{t-1}^{+}\right)}^{\lambda }\right)\right)}^{2}}}\\ \;=\frac{1}{n-1}\sum_{t=2}^{n}\frac{1}{1+\varphi \sqrt{{\left(\sum_{i=1}^{m}w_{i}({\left(\ln\;q_{t}^{+}\right)}^{\lambda }-{\left(\ln\;q_{it}^{+}\right)}^{\lambda })\right)}^{2}}+\rho \sqrt{{\left(\left(\sum_{i=1}^{m}w_{i}({\left(\ln\;q_{t}^{+}\right)}^{\lambda }-{\left(\ln\;q_{it}^{+}\right)}^{\lambda }))-(\sum_{i=1}^{m}w_{i}({\left(\ln\;q_{t-1}^{+}\right)}^{\lambda }-{\left(\ln\;q_{it-1}^{+}\right)}^{\lambda })\right)\right)}^{2}}}\\ \;=\frac{1}{n-1}\sum_{t=2}^{n}\frac{1}{\left(\begin{array}{l} 1+\varphi \sqrt{\sum_{i=1}^{m}\sum_{j=1}^{m}w_{i}w_{j}(\left({\left(\ln\;q_{t}^{+}\right)}^{\lambda }-{\left(\ln\;q_{it}^{+}\right)}^{\lambda })({\left(\ln\;q_{t}^{+}\right)}^{\lambda }-{\left(\ln\;q_{jt}^{+}\right)}^{\lambda }\right))}\\ +\rho \sqrt{\begin{array}{l} \sum_{i=1}^{m}\sum_{j=1}^{m}w_{i}w_{j}(\left({\left(\ln\;q_{t}^{+}\right)}^{\lambda }-{\left(\ln\;q_{it}^{+}\right)}^{\lambda })({\left(\ln\;q_{t}^{+}\right)}^{\lambda }-{\left(\ln\;q_{jt}^{+}\right)}^{\lambda }\right))\\ -\sum_{i=1}^{m}\sum_{j=1}^{m}w_{i}w_{j}(\left({\left(\ln\;q_{t-1}^{+}\right)}^{\lambda }-{\left(\ln\;q_{it-1}^{+}\right)}^{\lambda })({\left(\ln\;q_{t-1}^{+}\right)}^{\lambda }-{\left(\ln\;q_{jt-1}^{+}\right)}^{\lambda }\right)) \end{array}} \end{array}\right)} \end{array}$$ (27)

The following optimization issue results from the application of the gray correlation criteria to the interval combinatorial prediction model based on triangular fuzzy numbers and the GIOWLA operator:

$$\begin{array}{l} \max\gamma^{-},\max\gamma ,\max\gamma^{+}\\ \left\{ \begin{array}{c} \sum_{i=1}^{n}\omega_{i}=1\\ \omega_{i}\geq 0,i=1,2,\cdots ,n \end{array}\right. \end{array}$$ (28)

Preference coefficients *α*, *β*, *θ* are added in order to solve this nonlinear optimization problem: *γ*(*w*) = *αγ*^−^+*βγ*+*θγ*^+^. The gray correlation of combination prediction is represented by the symbol *γ*(*w*). The stronger the gray correlation, the more successful the combination prediction approach is, according to the gray correlation principle. It is possible to convert the gray correlation-based combination prediction model from a multi-objective optimization model to a single-objective optimization problem, which can be presented as follows:

$$\begin{array}{l} \max\gamma (w)=\max(\alpha \gamma^{-}+\beta \gamma +\theta \gamma^{+})\\ \left\{ \begin{array}{c} \sum_{i=1}^{n}\omega_{i}=1\\ \omega_{i}\geq 0,i=1,2,\cdots ,n\\ \alpha +\beta +\theta =1\\ 0\leq \alpha ,\beta ,\theta \leq 1 \end{array}\right. \end{array}$$ (29)

The relevance of left, center, and right ends of the triangle fuzzy number’s for the combination prediction is indicated by the preference coefficients *α*, *β*, and *θ*, respectively. The combination prediction value of each moment can be solved by using the Lingo software and the weight coefficients for the combination prediction can be obtained.

## 5. Experiment and analysis of results

### 5.1 Data collection

**Fig 1:**
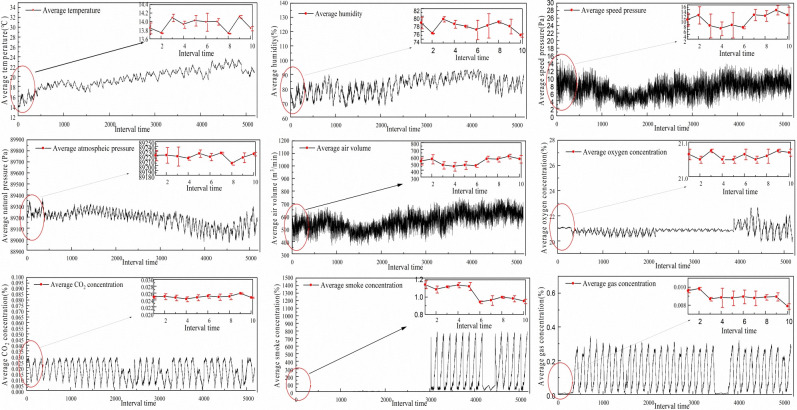
Nine original data sets.

In this study, air volume data was obtained from a coal mine tunnel in the Inner Mongolia Autonomous Region. As the mining depth increases, the structure of the mine ventilation system becomes increasingly complex leading to problems such as an increase in the content of harmful gases in the air, an increase in the ground temperature, and an increase in the amount of surging water. Sensor monitoring data can play a vital role in coal mine disaster warnings. This study utilized tunnel temperature, humidity, oxygen, velocity pressure, CO_2_, gas pressure, and dust as predictors of tunnel air volume. Sampling began at 0:00 each day for 36 days, from June 21, 2023, to July 26, 2023. Due to the variation in timing of sensor power-up and upload speed, the data for these nine factors were not available at the same time simultaneously. Therefore, the time interval for collecting time series data was set to 10 min, i.e., every 10 min as an interval. The maximum value of various data within 10 min was selected as the right endpoint of the interval and the minimum value as the left endpoint of the interval. This resulted in 5184 interval values for each factor time series, and the dataset is depicted in **Fig 1**. The data for this study was collected by the research team and does not involve human or animal subjects, therefore ethical review or approval is not required. All data were obtained in a legal and ethical manner, and appropriate measures were taken during data processing to ensure privacy and data security.

### 5.2 Data preprocessing results

**Fig 2:**
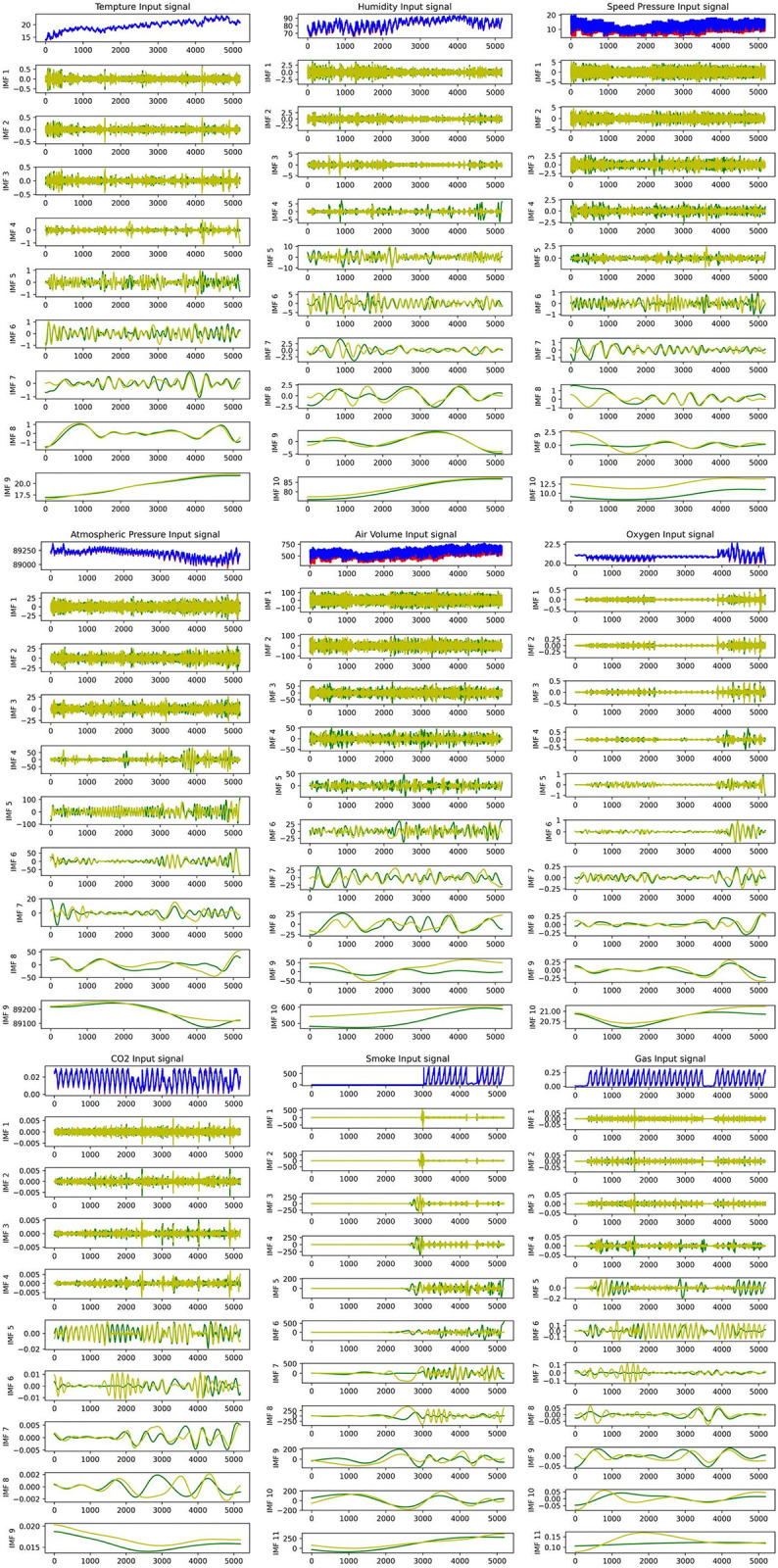
Modal decomposition diagram.

To more accurately estimate the tunnel air volume value and determine the underlying law of the air volume fluctuation, the time series of the mine tunnel air volume and the influencing factors are decomposed using the In-CEEMDAN model. **Fig 2** illustrates the decomposition results, which consist of one multivariate residual ***R*** component and several multivariate ***IMF*** components with frequencies ranging from high to low.

The time spectra show that ***IMF***1~***IMF***6 in each image reflects the fluctuation characteristics of the sequence of factors’, while ***IMF***7~***IMF***10(*or* ***IMF9***) indicates the trend characteristics of each factor, i.e., they represent original sequence’s trend for each factors. The fact that the multivariate ***IMF*** components vary up and down at around zero level which not change the asymmetry of the ***IMF*** waveforms, indicating that the original dataset is non-smooth and non-linear. On the other hand, time-frequency processing weakens the linearity of each decomposition component, which might enhance prediction performance.

**Fig 3:**
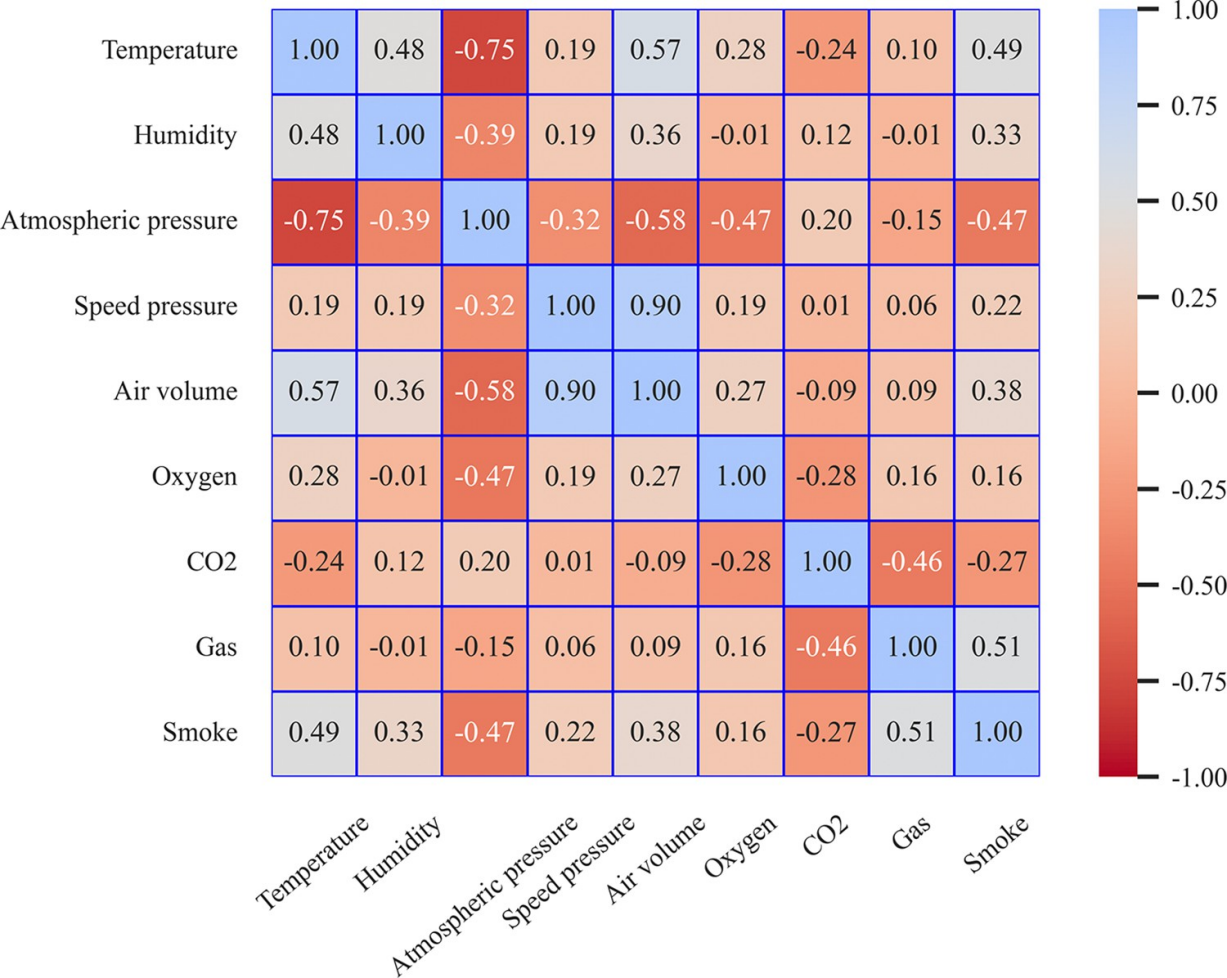
Correlation coefficient values for nine factors.

The Pearson correlation coefficient was used to quantitatively characterize the relationship between mine air volume, temperature, humidity, speed pressure, atmospheric pressure, oxygen, CO_2_, smoke, and gas. The results are shown in **Fig 3** and can be used to more effectively to screen out the meteorological and environmental factors which are high correlation with the tunnel air volume, while reducing dimensionality of the input features.

The Pearson correlation coefficient measures the magnitude of a linear correlation [38], with values ranging from -1 to 1. A coefficient value greater than 0 indicates a positive correlation while, a coefficient value = less than 0 represents a negative correlation. A correlation coefficient of 1 indicates perfect correlation, [0.8,1) indicates strong correlation, [0.5,0.8) indicates moderate correlation, [0.2,0.5) indicates weak correlation, and [0,0.2) indicates no correlation. As can be seen from the **Fig 3**, the air volume strongly correlates with velocity pressure, moderately correlate with air pressure, temperature, smoke, and gas, and demonstrates almost no correlation with humidity, oxygen, and CO_2_. Factors with above moderate correlation were considered based on Pearson’s quantitative correlation coefficient scale analysis. Therefore, velocity pressure, pressure, temperature, smoke, and gas can be used as input features to form a 6-dimensional multivariate time series with the air volume sequence.

**Fig 4:**
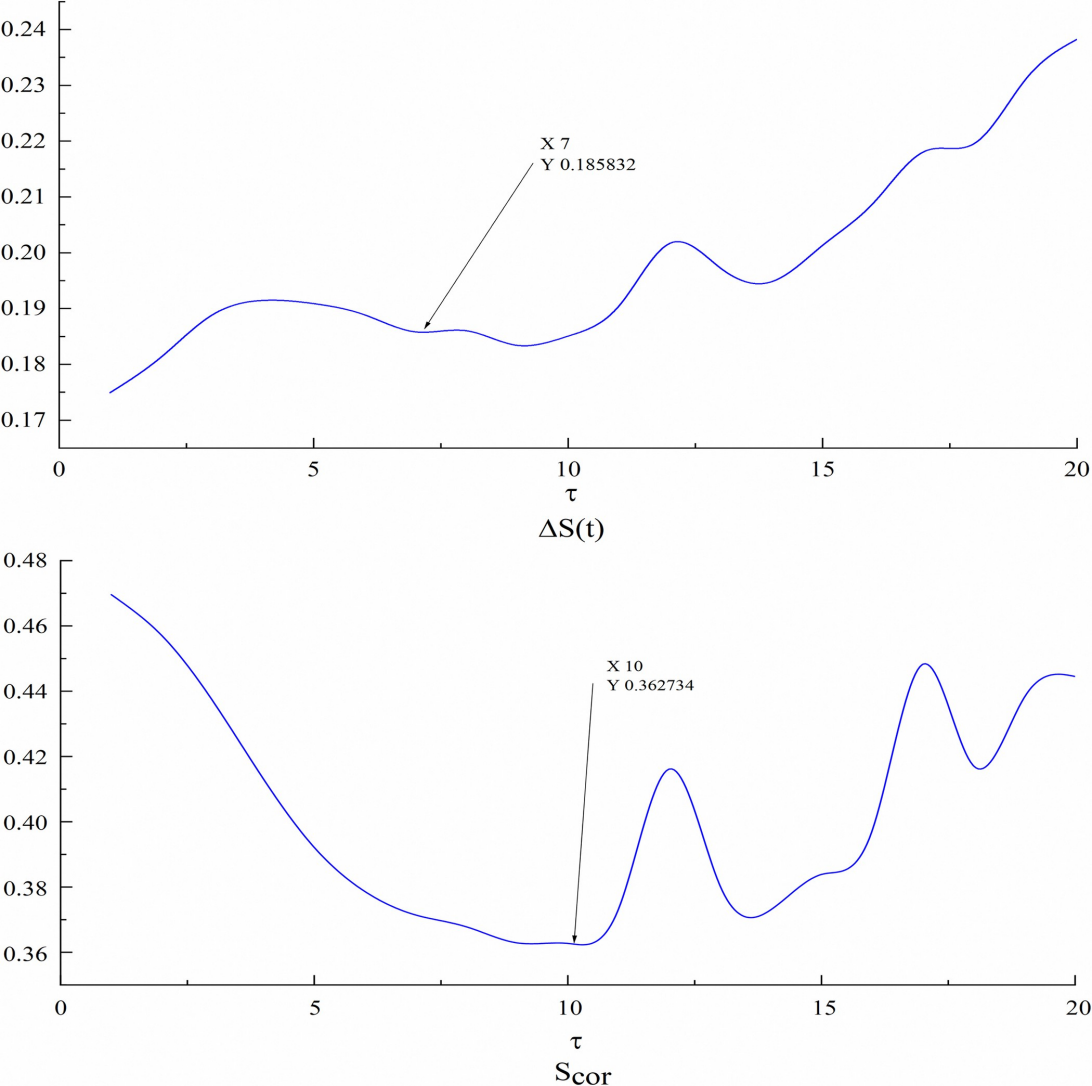
Embedding dimension and delay time of temperature.

The $\Delta \bar{S}$ curve and *S*_*cor*_ curve of the temperature can be calculated as shown in **Fig 4**.

It can be seen from the figure that the horizontal coordinates of the first local minimum point of $\Delta \bar{S}$ is 7, so the optimal delay time *τ*_*i*_ = 7, *S*_*cor*_ global minimum horizontal coordinate is 10, so the embedded window *τ*_*w*_ = 10, according to the formula *τ*_*w*_ = (*m*−1)*τ* can calculate the optimal phase space dimension to *m*_*i*_ = 2. In the same way, estimated optimal embedding dimension *m*_*i*_ and time delay *τ*_*i*_ for the multivariate time series, and the results are shown in **Table 1**.

**Table 1:** Optimal embedding dimension and time delay.

Influencing factor	*m*_*i*_	*τ*_*i*_
Air volume	3	8
Speed pressure	5	4
Pressure	2	6
Temperature	2	10
Smoke	4	8
Gas	7	4

The wolf method calculated the maximum Lyapunov exponents (including the left and right endpoints of the interval) of the above six-factor interval series [39]. *λ*_*IP*_ is 0.1149, and *λ*_*rp*_ is 0.0938 for temperature; *λ*_*IP*_ is 0.2327, and *λ*_*rp*_ is 0.3739 for pressure; *λ*_*IP*_ is 0.6906, and *λ*_*rp*_ is 0.4487 for velocity pressure; *λ*_*IP*_ is 1.4421, and *λ*_*rp*_ is 1.0811 for air volume; *λ*_*IP*_ is 0.0026 and *λ*_*rp*_ is 0.0078 for smoke; *λ*_*IP*_ is 0.0325 and *λ*_*rp*_ is 0.0323 for gas. All *λ*_*IP*_ and *λ*_*rp*_ are more significant than 0, indicating that the multivariate time series of mine tunnel air volume has chaotic properties.

### 5.3 Result analysis

This section demonstrates three experiments. Experiment Ⅰ compares different single prediction models before and after data preprocessing to demonstrate the superiority of data processing. Experiment Ⅱ compares different single-item prediction models before and after phase space reconstruction to demonstrate the superiority of phase space reconstruction. Lastly, Experiment Ⅲ compares the combined prediction model and the single prediction model to highlight the superiority of the combined prediction.

In both Experiment Ⅰ and Experiment Ⅱ, the type of predicted data is in the form of intervals, so different evaluation indexes are selected and represented using the form of intervals to comprehensively evaluate and compare the predictive performance of the models. Mean absolute error (MAE), root mean square error (RMSE), mean absolute percentage error (MAPE), average relative variance (ARV), and overlap (OL) are used as evaluation metrics for single prediction models to assess the prediction accuracy of the lower and upper bounds of the interval air volume. MAE can better express the reality of the prediction error; the closer the value of MAE is to 0, the better the model’s performance. RMSE reflects the deviation of the predicted data from the actual data. MAPE considers the ratio of the error to the original value to reflect the accuracy of the prediction. Due to the interval structure of the predicted data, ARV is used to measure the overall accuracy of the fitted and predicted air volume, and OL is utilized to characterize the degree of coverage between the predicted and actual intervals. The specific calculations for these evaluation metrics are as follows:

$$MAE_{i}^{-}=\frac{1}{N}\sum_{t=1}^{N}\left|q_{t}^{-}-q_{it}^{-}\right|,\;MAE_{i}^{+}=\frac{1}{N}\sum_{t=1}^{N}\left|q_{t}^{+}-q_{it}^{+}\right|$$ (30)

$$RMSE_{i}^{-}=\sqrt{\frac{1}{N}\sum_{t=1}^{N}{\left(q_{t}^{-}-q_{it}^{-}\right)}^{2}},\;RMSE_{i}^{+}=\sqrt{\frac{1}{N}\sum_{t=1}^{N}{\left(q_{t}^{+}-q_{it}^{+}\right)}^{2}}$$ (31)

$$MAPE_{i}^{-}=\frac{1}{N}\sum_{t=1}^{N}\left|\frac{q_{t}^{-}-q_{it}^{-}}{q_{t}^{-}}\right|\times 100\%,\;MAPE_{i}^{+}=\frac{1}{N}\sum_{t=1}^{N}\left|\frac{q_{t}^{+}-q_{it}^{+}}{q_{t}^{+}}\right|\times 100\%$$ (32)

$$ARV_{i}^{Inter}=\frac{\sum_{t=1}^{N}{\left(q_{t}^{-}-q_{it}^{-}\right)}^{2}+\sum_{t=1}^{N}{\left(q_{t}^{+}-q_{it}^{+}\right)}^{2}}{\sum_{t=1}^{N}{\left(q_{t}^{-}-\bar{q^{-}}\right)}^{2}+\sum_{t=1}^{N}{\left(q_{t}^{+}-\bar{q^{+}}\right)}^{2}}$$ (33)

$$OverLap_{i}=\left(\sum_{t=1}^{N}\frac{\left|\min(q_{t}^{-},q_{it}^{-})-\max(q_{t}^{+},q_{it}^{+})\right|}{\left|\max(q_{t}^{-},q_{it}^{-})-\min(q_{t}^{+},q_{it}^{+})\right|}\right)/N$$ (34)

#### 5.3.1 Experiment I

**Fig 5:**
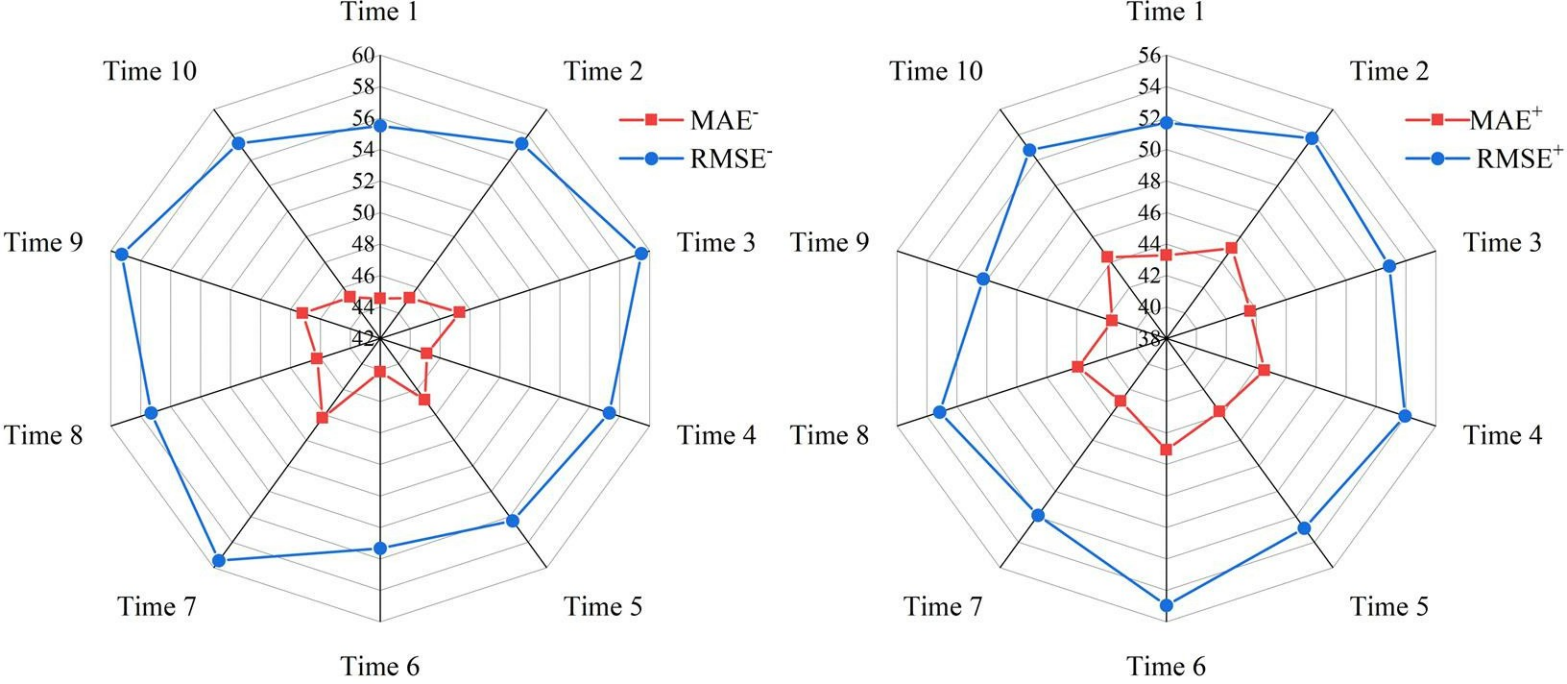
Results of 10 runs of LSTM.

Four neural network models(CNN, LSTM, GRU, and SNN) were developed as benchmark models to effectively illustrate the predictive performance of data preprocessing. These models were compared which using In-CEEMDAN. To ensure the accuracy of the results, all prediction algorithms used in this study are run 10 times. Take the LSTM example and analyze it using MAE, RMSE for statistical tests to see the results of 10 runs, and the results are shown in **Table 2** and **Fig 5**.

**Table 2:** Results of 10 runs of LSTM.

Times	*MAE* ^−^	*MAE* ^+^	*RMSE* ^−^	*RMSE* ^+^
1	44.5223	43.2885	55.5094	51.6965
2	45.1776	45.5764	57.2997	53.7214
—	—	—	—	—
9	47.1747	41.6218	59.2797	50.2376
10	45.2416	44.3724	57.3219	52.7912

**Fig 6:**
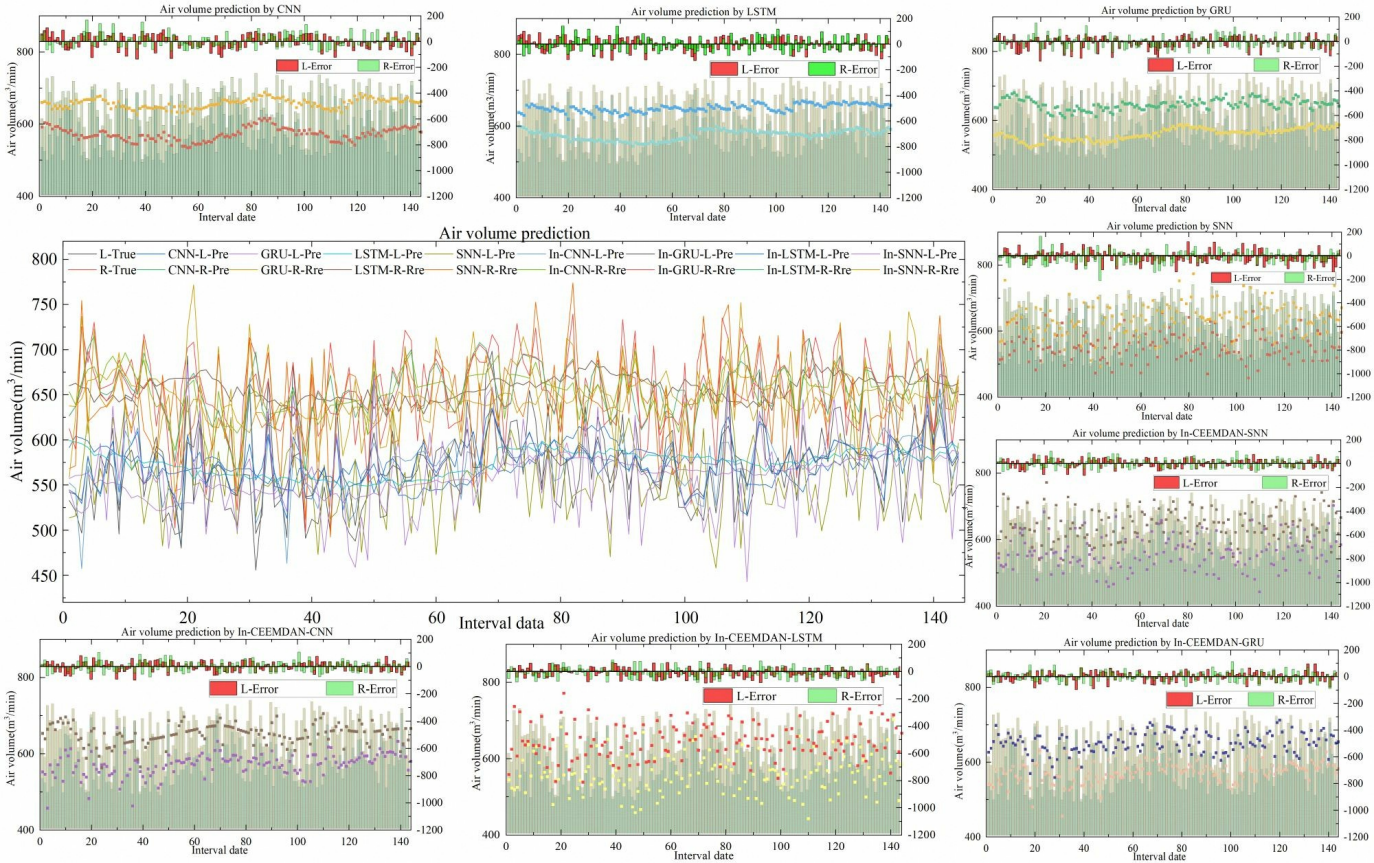
Comparison of prediction results before and after data preprocessing.

As can be seen by **Fig 5**, the overall MAE and RMSE of the prediction results for 10 runs do not change much. Therefore, the average of the results of 10 runs was taken for subsequent experimental analysis. **Table 3** presents the detailed comparative results while additional predicted specific results are shown in **Fig 6**.

**Table 3:** Comparison of error indicators of prediction results before and after data preprocessing.

Models	*MAE* ^−^	*MAE* ^+^	*MAPE* ^−^	*MAPE* ^+^	*RMSE* ^−^	*RMSE* ^+^	*ARV*	*OL*
CNN	48.5321	45.5421	8.326	7.027	57.6864	55.7532	1.0732	0.3621
LSTM	46.4461	44.3524	8.014	6.832	56.6621	53.6352	1.0362	0.3795
GRU	45.1264	44.2271	7.965	6.776	55.1457	54.7785	1.0675	0.3841
SNN	45.0558	45.3481	7.803	6.943	56.1385	57.6045	1.1594	0.4332
In-CEEMDAN-CNN	33.2101	33.9035	5.777	5.256	40.3157	40.1567	0.5803	0.4607
In-CEEMDAN-LSTM	32.2947	32.6957	5.664	5.099	38.0452	39.0698	0.5329	0.4345
In-CEEMDAN-GRU	31.3834	31.6842	5.492	4.913	37.0906	38.1133	0.5068	0.5103
In-CEEMDAN-SNN	32.6551	33.1726	5.761	5.127	38.9475	40.4906	0.5656	0.4935

(1) The predictive performance of each model is measured using five statistical metrics, where MAE, MAPE, and RMSE measure the degree of predictive accuracy. The lower these metrics are, the more accurate the model’s predictions are. For the tunnel air volume data, the four single prediction models are preprocessed by In-CEEMDAN noise reduction, and the MAE values of the left endpoints are reduced by 15.322, 14.1514, 13.743, and 12.4007; the left endpoint MAPE values decreased by 2.549, 2.35, 2.473, and 2.042; the left endpoint RMSE values decreased by 17.3707, 18.6169, 18.0551, and 17.191. The right endpoint MAE values decreased by 11.6386, 11.6567, 12.5429, and 12.1755; the right endpoint MAPE values decreased by 1.771, 1.733, 1.863, and 1.816; and the right endpoint RMSE values decreased by 15.5965, 14.5654, 16.6652, and 17.1139. The values of MAE, MAPE, and RMSE after processing are smaller than the predicted values without data preprocessing, proving that the data preprocessing method significantly improves the prediction accuracy in the proposed model.

(2) The ARV metric indicates the extent to which the predicted values of the left and right endpoints of the air volume interval are dispersed from the mean values of the left and right endpoints. A small degree of dispersion resulting in a slight variance. The four single-item prediction models were preprocessed with noise reduction, the ARV values were reduced by 0.4929, 0.5033, 0.5607, and 0.5938. This demonstrates that the fluctuations of the model’s predicted values after data preprocessing are more closer to the true values.

(3) The OL metric measures the average proportion of actual intervals covered by the fitted intervals, with all coverage measures are between 0 and 1, with higher values being more appropriate. The four single-item prediction models are pre-processed with In-CEEMDAN noise reduction, and the OL values are improved by 0.0986, 0.055, 0.1262, and 0.0603. This demonstrates that the predictive model after data processing are able to cover more of the true values.

Based on Experiment Ⅰ, it is evident that the prediction method after In-CEEMDAN processing can produce better prediction results. This demonstrates the effective performance of In-CEEMDAN processing in nonlinear pattern modeling. Because In-CEEMDAN can separate and extract multiple frequency components in the nonlinear pattern of the left and right endpoints of the interval sequence, the learning ability and prediction accuracy of the four prediction models for the highly irregular time series of duct air volume values are significantly improved by reducing the noise of the raw data.

#### 5.3.2 Experiment II

**Fig 7:**
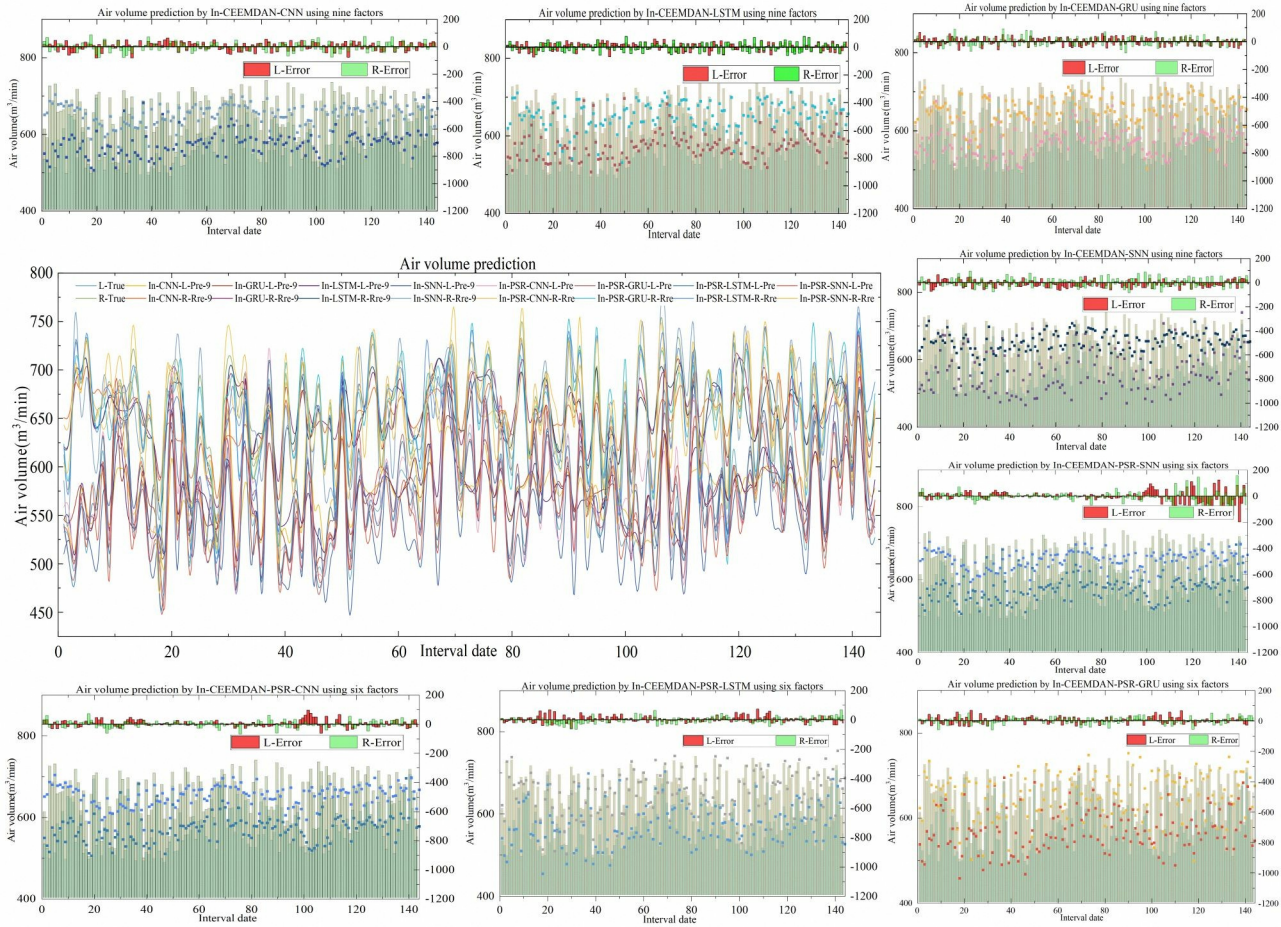
Comparison of prediction results before and after phase space reconstruction.

To validate the effectiveness of the prediction model proposed in this study, which uses neural network fusion of multi-sensor information, a tunnel air volume prediction method based on nine variables (tunnel air volume, temperature, pressure, velocity pressure, gas, smoke, oxygen, CO_2_, and humidity) is compared with a method using six variables (tunnel air volume, temperature, pressure, velocity pressure, gas, and smoke) after phase space reconstruction. **Table 4** provides detailed comparison results, and **Fig 7** presents the specific results of the additional predictions.

**Table 4:** Comparison of error metrics of prediction results before and after phase space reconstruction.

Models	*MAE* ^−^	*MAE* ^+^	*MAPE* ^−^	*MAPE* ^+^	*RMSE* ^−^	*RMSE* ^+^	*AVG*	*OL*
In-CEEMDAN-CNN-9	26.6232	27.1593	4.659	4.229	31.9557	33.7829	0.3877	0.5886
In-CEEMDAN-LSTM-9	25.6497	26.5677	4.487	4.118	30.0016	31.8856	0.3435	0.5213
In-CEEMDAN- GRU-9	25.3257	25.8722	4.406	4.057	29.6532	31.9862	0.3409	0.5386
In-CEEMDAN- SNN-9	27.6108	28.0981	4.842	4.335	32.3681	32.0713	0.3784	0.5104
In-CEEMDAN-PSR-CNN	17.2039	18.0017	3.064	2.782	23.7303	23.426	0.1992	0.7613
In-CEEMDAN-PSR-LSTM	17.2797	17.3619	3.091	2.684	22.7728	22.7819	0.1859	0.7333
In-CEEMDAN-PSR-GRU	16.7627	17.1736	2.975	2.647	21.9774	22.1136	0.1742	0.7481
In-CEEMDAN-PSR-SNN	18.2047	17.2362	3.342	2.653	23.0595	21.6144	0.1790	0.6593

In-CEEMDAN-CNN-9: Nine input variables and preprocessed the data using In-CEEMDAN as input values to the CNN; In-CEEMDAN-PSR-CNN: Six input variables were obtained using phase space reconstruction and data preprocessing was performed using In-CEEMDAN as input values to the CNN.

(1) As can be seen from **Table 4**, the four single prediction models, In-CEEMDAN-CNN-9, In-CEEMDAN-LSTM-9, In-CEEMDAN-GRU-9, and In-CEEMDAN-SNN-9, due to significant number of input variables, the values of the evaluation indexes at the left endpoints and the right endpoints of the interval sequence of predicted air volume values changes compared to the models from Table 2 (In-CEEMDAN-CNN, In-CEEMDAN-LSTM, In-CEEMDAN-GRU, and In-CEEMDAN-SNN. CNN). Specifically, the left endpoint MAE values decreased by 6.5869, 6.645, 6.0577, and 5.0443; the left endpoint MAPE values decreased by 1.118, 1.177, 1.086, and 0.919; and the left endpoint RMSE values decreased by 8.36, 8.0436, 7.4374, and 6.5794. The right endpoint MAE values decreased by 6.7442, 6.128, 5.812, and 5.0745; the right endpoint MAPE values decreased by 1.027, 0.981, 0.856, and 0.792; and the right endpoint RMSE values decreased by 6.3738, 7.1842, 6.1271, and 8.4193. ARVs decreased by 0.1926, 0.1894, 0.1659, and 0.1872; OLs increased by 0.1279, 0.0868, 0.0283, and 0.0169. The a single input variable prediction models utilizes the time series of historical air volume intervals to make predictions without considering the factors related to the air volume. Therefore, it is difficult to obtain satisfactory prediction results and less reliable results. On the other hand, the multi-input variable prediction model incorporates the influencing factors related to the air volume, leading to more accurate mapping relationship between the inputs and outputs. Compared with the prediction model with a single input variable, the prediction model with nine input variables further improves the convergence effect and prediction accuracy.

(2) The predictive model using nine input variables is also compared with the model with six input variables obtained after phase space reconstruction. The results indicated that the left endpoint MAE values decreased by 9.4193, 8.37, 8.563, and 9.4061; the left endpoint MAPE values decreased by 1.595, 1.396, 1.431, and 1.5; and the left endpoint RMSE values decreased by 8.2254, 7.2288, 7.6758, and 9.3086. The right endpoint MAE values decreased by 9.1576, 9.2058, 8.6986, and 10.8619; the right endpoint MAPE values decreased by 1.447, 1.434, 1.41, and 1.682; and the right endpoint RMSE values decreased by 10.3569, 9.1037, 9.8726, and 10.4569. ARVs decreased by 0.1885, 0.1576, 0.1667, and 0.1994; OLs increased by 0.1727, 0.212, 0.2095, and 0.1489. The prediction model with six input variables performs better than the model with nine input variables. The predictive model with nine input variables has a worse model convergence performance than the predictive model constructed with six correlation input variables due to the introduction of more influencing factors. This suggests that inputs with strong correlation help to improve the prediction performance of different single-item prediction models, whereas the inputs of weakly correlated or uncorrelated factors, on the contrary, reduce their prediction performance.

(3) Comparing the four different single prediction algorithms after phase space reconstruction, In-CEEMDAN-PSR-GRU has the smallest MAE, MAPE, which indicates that this algorithm has the best prediction performance and higher prediction accuracy. However, In-CEEMDAN-PSR-CNN has the highest OL, indicating that the algorithm has the highest coverage of the prediction results, and the overall fluctuation trends are closer to the actual values. In other words, no single algorithm can completely optimize the prediction results, and each algorithm has prediction strengths and weaknesses.

According to Experiment Ⅱ, it can be concluded that by comparing the models with different input data demonstrates that multifactor prediction model which utilizes the input variables input variables determined after correlation analysis and phase space reconstruction, significantly improve the prediction accuracy of the air volume in the coal mine tunnel.

#### 5.3.3 Experiment Ⅲ

Experiment Ⅲ combines the predictions performed by the three separate prediction techniques and compares to the prediction results with single prediction methods.

According to definition 2, the midpoint of the interval number is selected as the midpoint of the triangular fuzzy number The interval number is then converted into the triangular fuzzy number. The left precision, middle precision, and right precision of the triangular fuzzy number of the three single prediction methods are calculated at each moment based on the Eqs (15), (16) and (17), along with two-dimensional array induced by each method. The calculation results are presented in **Table 5**.

**Table 5:** Air volume single item predictive accuracy.

Induced two-dimensional arrays		1	2	—	144
Lift endpoint	$<u_{1t}^{-},q_{1t}^{-}>$	<0.952,558.52>	<0.943,551.10>	—	<0.966,531.44>
	$<u_{2t}^{-},q_{2t}^{-}>$	<0.985,541.02>	<0.957,499.18>	—	<0.958,526.97>
	$<u_{3t}^{-},q_{3t}^{-}>$	<0.989,538.52>	<0.957,498.81>	—	<0.993,546.61>
	$<u_{4t}^{-},q_{4t}^{-}>$	<0.990,537.99>	<0.994,524.54>	—	<0.972,565.83>
Mid endpoint	<*u*_1*t*_,*q*_1*t*_>	<0.951,600.82>	<0.921,599.73>	—	<0.992,599.49>
	<*u*_2*t*_,*q*_2*t*_>	<0.985,581.31>	<0.974,540.99>	—	<0.996,607.19>
	<*u*_3*t*_,*q*_3*t*_>	<0.985,581.04>	<0.983,546.34>	—	<0.989,611.19>
	<*u*_4*t*_,*q*_4*t*_>	<0.977,586.11>	<0.979,567.24>	—	<0.978,617.76>
Right endpoint	$<u_{1t}^{+},q_{1t}^{+}>$	<0.950,643.11>	<0.901,648.35>	—	<0.987,667.53>
	$<u_{2t}^{+},q_{2t}^{+}>$	<0.985,621.59>	<0.988,582.81>	—	<0.957,687.41>
	$<u_{3t}^{+},q_{3t}^{+}>$	<0.982,623.55>	<0.993,593.87>	—	<0.974,675.76>
	$<u_{4t}^{+},q_{4t}^{+}>$	<0.965,634.22>	<0.966,609.94>	—	<0.984,669.69>

The results of the single prediction methods are dimensionless. According to **Definition 3**, the gray correlations of the left endpoints, right endpoints, and midpoint sequences of the three single prediction methods can be calculated. The calculation results are depicted in **Table 6**.

**Table 6:** Error correction of the first four single predictions left, right point and the maximum of the midpoint-minimum closeness.

Single prediction method	$\gamma_{i}^{-}$	*γ*_*i*_	$\gamma_{i}^{+}$
In-CEEMDAN-CNN	0.8910	0.9102	0.8843
In-CEEMDAN-LSTM	0.8649	0.9065	0.8812
In-CEEMDAN-GRU	0.8663	0.9031	0.8823
In-CEEMDAN-SNN	0.8756	0.9017	0.8902

Different values of *λ* will result in different generalized induced ordered weighting operators, producing varied combined air volume prediction values. In this research, we design an interval based combined air volume prediction model. The process of calculating the combined prediction of air volume is illustrated with the left endpoint as follows: ${\hat{q}}_{1}^{-}=f_{w}(\langle {\tilde{\delta }}_{11},q_{11}^{-}\rangle ,\langle {\tilde{\delta }}_{21},q_{21}^{-}\rangle ,\langle {\tilde{\delta }}_{31},q_{31}^{-}\rangle ,\langle {\tilde{\delta }}_{41},q_{41}^{-}\rangle )$ $=f_{w}(\langle 0.952,558.52\rangle ,\langle 0.985,541.02\rangle ,\langle 0.989,538.52\rangle ,\langle 0.990,537.99\rangle )$ =exp{(∑i=14wi(lnait)λ)1λ} =exp{((ln537.99)λw1+(ln538.52)λw2+(ln541.02)λw3+(ln558.52)λw4)1λ}.

In similar way, the predicted values of the combined air volume at the left, center, and right endpoints at other moments can be obtained as well.

According to Eq (29), *Lingo* software is used to give the weight coefficient values of individual predictions when the preference coefficients are *α* = 3/10, *β* = 3/5, *θ* = 1/10 and *α* = 1/5, *β* = 3/4, *θ* = 1/20. The weight coefficients *w*_1_, *w*_2_, *w*_3_, and *w*_4_ for solving the combined air volume value prediction model for each of the above parameters at different values are demonstrated in **Table 7**.

**Table 7:** Each parameter values and corresponding power system values.

Parameter value		*w*_1_	*w*_2_	*w*_3_	*w*_4_
*λ* = 1	*α* = 3/10, *β* = 3/5, *θ* = 1/10	0.73649	0.20565	0.04584	0.01202
	*α* = 1/5, *β* = 3/4, *θ* = 1/20	0.72024	0.20926	0.05043	0.02007
*λ* = 2	*α* = 3/10, *β* = 3/5, *θ* = 1/10	0.67227	0.23977	0.07545	0.01251
	*α* = 1/5, *β* = 3/4, *θ* = 1/20	0.66314	0.24917	0.05928	0.02841
*λ* = 5	*α* = 3/10, *β* = 3/5, *θ* = 1/10	0.57760	0.25731	0.10728	0.05781
	*α* = 1/5, *β* = 3/4, *θ* = 1/20	0.57886	0.25371	0.09898	0.06845

**Fig 8:**
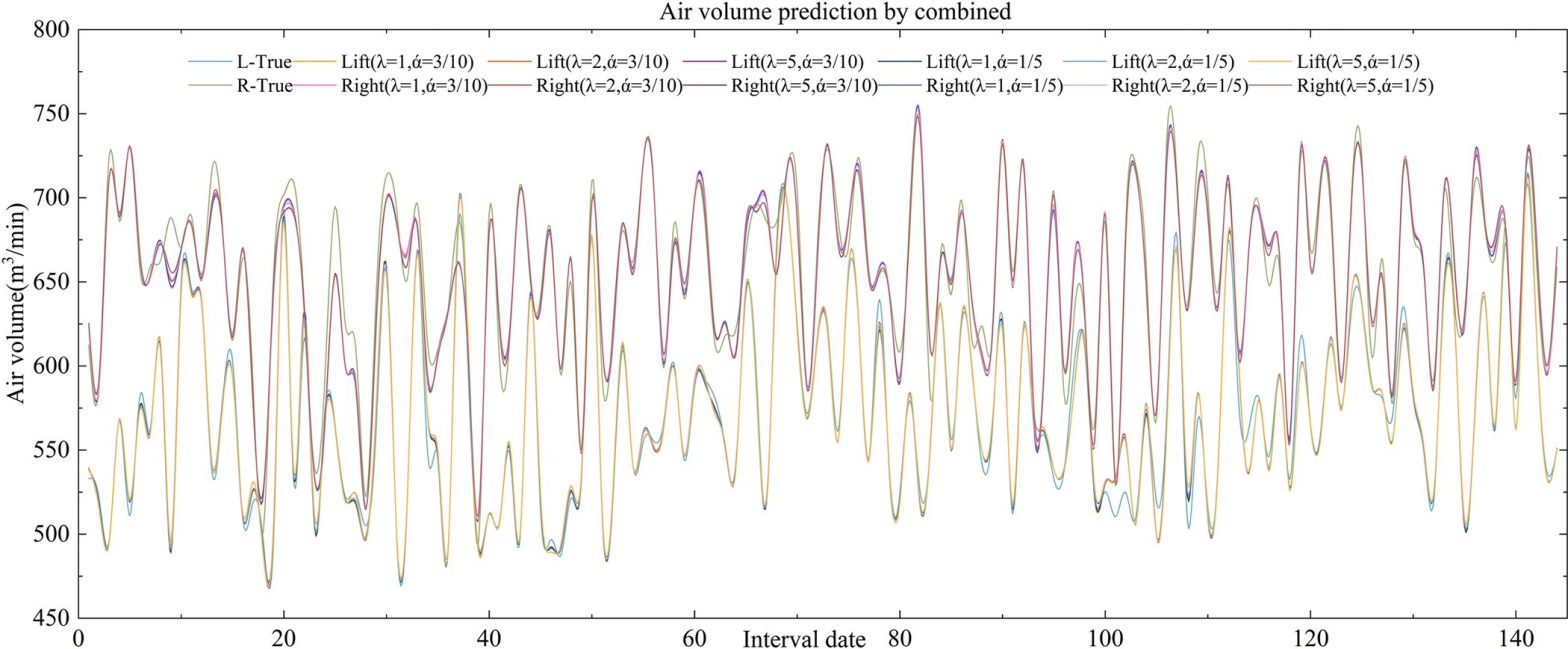
Combined prediction of air volume.

Based on the results of the weighting factor *λ* in **Table 7**, the predicted values of the combined air volume at each moment are calculated for the cases *α* = 3/10, *β* = 3/5, *θ* = 1/10, and *α* = 1/5, *β* = 3/4, *θ* = 1/20, and the results are shown in **Tables 8** and 9 as well as in **Fig 8**.

**Table 8:** Error correction of different values of *λ* triangular fuzzy combination of air volume prediction values under different values(*α* = 3/10, *β* = 3/5, *θ* = 1/10).

Parameter value	1	2	—	144
Actual data	[532.92,572.71,612.50]	[521.46,555.67,589.87]	—	[550.21,604.51,658.82]
*λ* = 1	[538.18,581.56,623.87]	[517.99,551.03,587.09]		[550.43,605.91,668.98]
*λ* = 2	[538.28,581.73,624.13]	[516.37,551.61,588.16]		[551.19,605.77,669.64]
*λ* = 5	[538.16,582.79,625.32]	[515.03,554.23,592.33]		[550.78,606.24,670.69]

**Table 9:** Error correction of different values of *λ* triangular fuzzy combination of air volume prediction values under different values(*α* = 1/5, *β* = 3/4, *θ* = 1/20).

Parameter value	1	2	—	144
Actual data	[532.92,572.71,612.50]	[521.46,555.67,589.87]	—	[550.21,604.51,658.82]
*λ* = 1	[538.15,581.74,624.07]	[517.76,551.49,587.76]	—	[550.36,605.98,669.14]
*λ* = 2	[538.16,581.96,624.24]	[516.50,552.71,588.99]	—	[550.99,605.80,669.47]
*λ* = 5	[540.52,585.63,628.33]	[517.56,557.33,595.49]	—	[553.02,609.12,673.75]

The combination prediction interval air volume model was evaluated using the combined weighted mean absolute error (CWMAE), combined weighted root of mean Squares error (CWRMSE), and combined weighted mean absolute percentage error (CWMAPE). The results of calculations are as follows:

$$CWMAE=\frac{\alpha }{N}\sum_{t=1}^{N}\left|q_{t}^{-}-{\hat{q}}_{t}^{-}\right|+\frac{\beta }{N}\sum_{t=1}^{N}\left|q_{t}-{\hat{q}}_{t}\right|+\frac{\theta }{N}\sum_{t=1}^{N}\left|q_{t}^{+}-{\hat{q}}_{t}^{+}\right|$$ (35)

$$CWRMSE=\alpha \sqrt{\frac{1}{N}\sum_{t=1}^{N}{\left(q_{t}^{-}-{\hat{q}}_{t}^{-}\right)}^{2}}+\beta \sqrt{\frac{1}{N}\sum_{t=1}^{N}{\left(q_{t}-{\hat{q}}_{t}\right)}^{2}}+\theta \sqrt{\frac{1}{N}\sum_{t=1}^{N}{\left(q_{t}^{+}-{\hat{q}}_{t}^{+}\right)}^{2}}$$ (36)

$$CWMAPE=(\frac{\alpha }{N}\sum_{t=1}^{N}\left|\frac{q_{t}^{-}-{\hat{q}}_{t}^{-}}{q_{t}^{-}}\right|+\frac{\beta }{N}\sum_{t=1}^{N}\left|\frac{q_{t}-{\hat{q}}_{t}}{q_{t}}\right|+\frac{\theta }{N}\sum_{t=1}^{N}\left|\frac{q_{t}^{+}-{\hat{q}}_{t}^{+}}{q_{t}^{+}}\right|)\times 100\%$$ (37)

Where *N* is the number of data and *α*, *β*, *θ* is the preference coefficient.

Eqs (35)(36)(37) can be used to determine CWMAE, CWRMSE, and CWMAPE for the expected air volumes of *α* = 3/10, *β* = 3/5, *θ* = 1/10 and *α* = 1/5, *β* = 3/4, *θ* = 1/20, for each of the three distinct prediction methods and interval combinations. **Table 10** presents the computations’ findings.

**Table 10:** Error indicators for single and combined prediction methods.

	*α* = 3/10, *β* = 3/5, *θ* = 1/10			*α* = 1/5, *β* = 3/4, *θ* = 1/20		
	CWMAE	CWRMSE	CWMAPE	CWMAE	CWRMSE	CWMAPE
In-CEEMDAN-PSR-CNN	14.8349	19.5370	2.4977	14.1829	18.5115	2.3773
In-CEEMDAN-PSR-LSTM	14.0004	18.1969	2.3619	13.1744	17.0522	2.2103
In-CEEMDAN-PSR-GRU	14.4465	18.0219	2.4235	13.8366	17.0228	2.3102
In-CEEMDAN-PSR-SNN	15.3065	18.9176	2.5393	14.4674	17.9842	2.3883
Combined prediction (*λ* = 1)	5.0384	6.9909	1.5204	5.1497	6.8889	1.5103
Combined prediction (*λ* = 2)	5.1695	7.0042	1.4801	5.1002	7.0260	1.4845
Combined prediction(*λ* = 5)	5.4539	7.3078	1.4756	5.5011	7.3425	1.4836

**Table 10** shows that the interval combination type air volume prediction model proposed in this research, with *λ* and varying preference coefficients values, CWMAE, CWRMSE, and CWMAPE values are significantly smaller than the three single-item prediction methods. This suggests that the model can significantly increase the prediction accuracy of the air volume values. However, as the value of *λ* increases, the prediction error accuracy further increases, which is due to the change in the weight coefficients. When the value of *λ* is increased from 1 to 5, the weight of the predicted value that is closest to the actual value decreases, and the weight of the predicted value that differs from the actual value increases, which leads to an increase in the combined prediction error.

In this study, only the values of the preference coefficients of the two groups are compared. A sensitivity analysis can be performed on the preference coefficients to better analyze the impact on the accuracy of the model predictions when the preference coefficients take other values.

**Fig 9:**
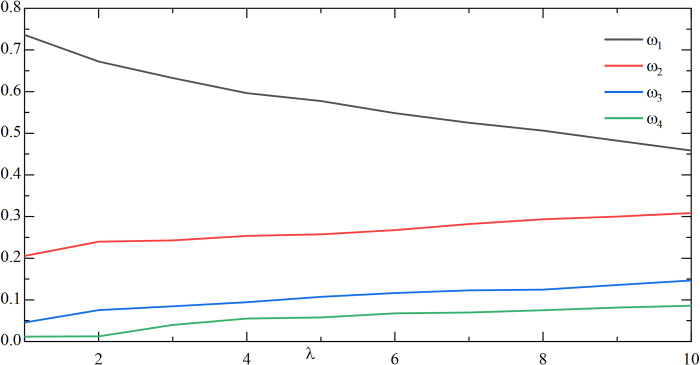
The effect of changes in *λ* on the weighting factor(*α* = 3/10, *β* = 3/5, *θ* = 1/10).

**Fig 10:**
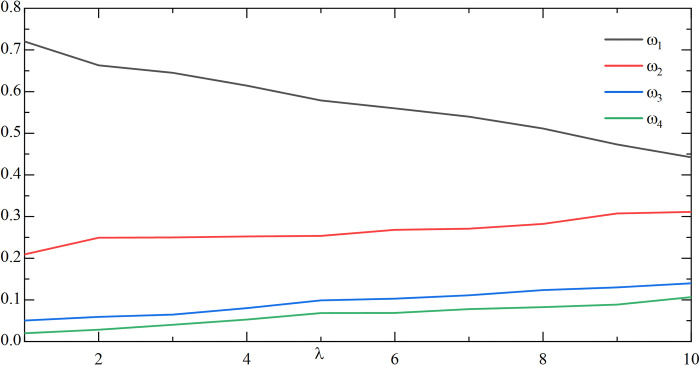
The effect of changes in *λ* on the weighting factor(*α* = 1/5, *β* = 3/4, *θ* = 1/20).

In order to evaluate the effect of the change of *λ* on the weighting coefficient *ω*_1_, *ω*_2_, *ω*_3_, *ω*_4_ in the combination prediction, the preference coefficient *α*, *β*, *θ* can be fixed initially. As an example, by analyzing the study of the two values of *α*, *β*, *θ*, the following **Figs 9** and **10** can be obtained:

As can be seen from **Figs 9** and **10**, with fixed preference coefficients *α*,*β*,*θ*, the change in *λ* has a large impact on *ω*_1_. The impact on *ω*_2_, *ω*_3_, and *ω*_4_ is relatively small and fluctuates only within a small range. By observing **Table 10**, it is found that the change of *λ* has a certain influence on CWMAE, CWRMSE, and CWMAPE, and the prediction accuracy gradually becomes larger as *λ* becomes larger, so the value of *λ* should not be taken too large.

To obtain the effect of the preference coefficient *α*, *β*, *θ* on the combination prediction weight coefficient *ω*_1_, *ω*_2_, *ω*_3_, *ω*_4_, the value of *λ* should be fixed, followed by fixing one of the preference coefficients *α*,*β*,*θ* and varying the remaining two. Through the above analysis of *λ*, it was obtained that the change in *λ* does not have much effect on the model prediction accuracy. For the convenience of calculation, it is sufficient to choose one value of *λ*. In this study, *λ* = 1 is selected.

**Fig 11:**
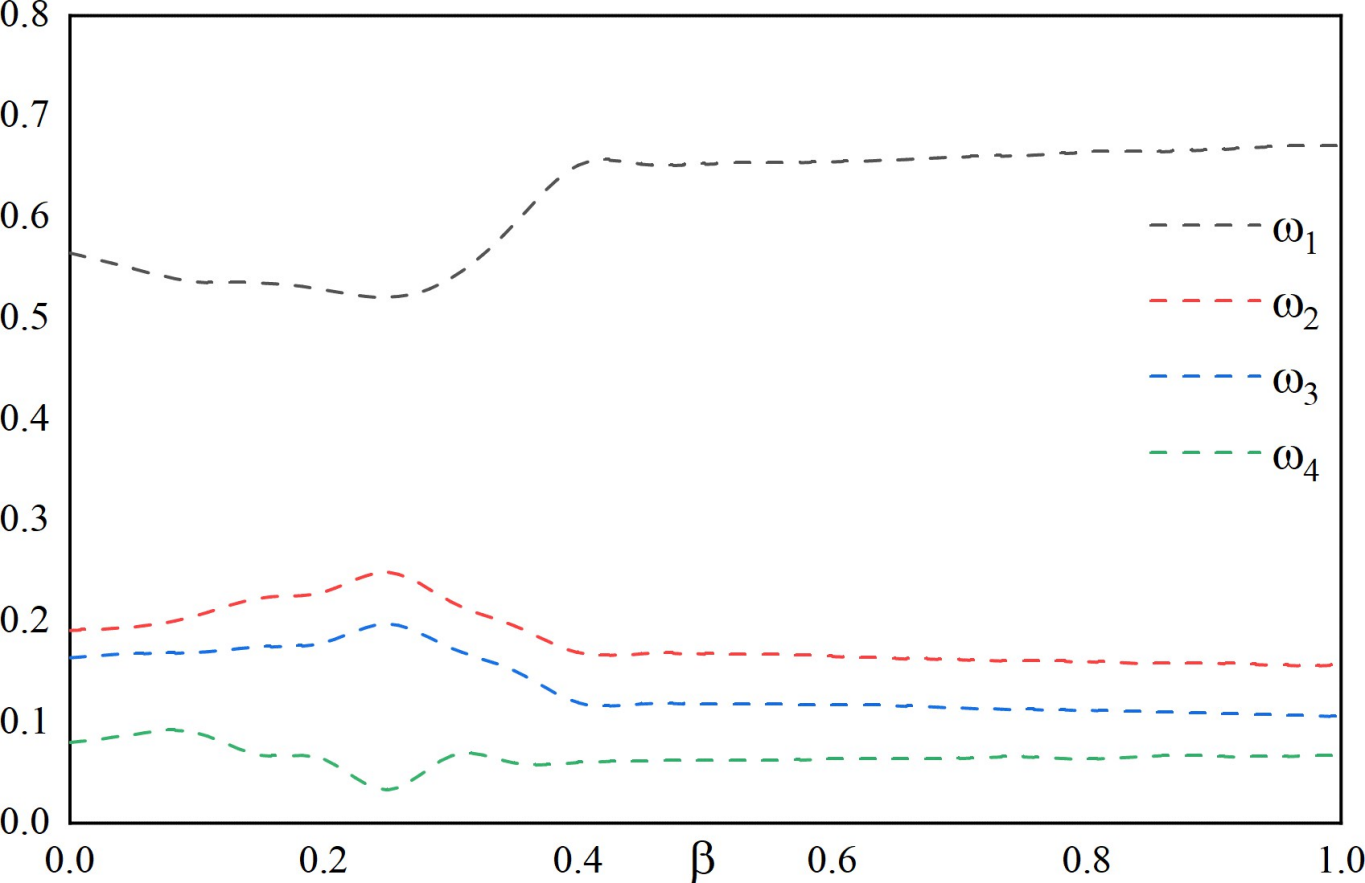
Effect of changes in *β* on the weighting factor (fixed *α* = 0, *λ* = 1).

**Fig 12:**
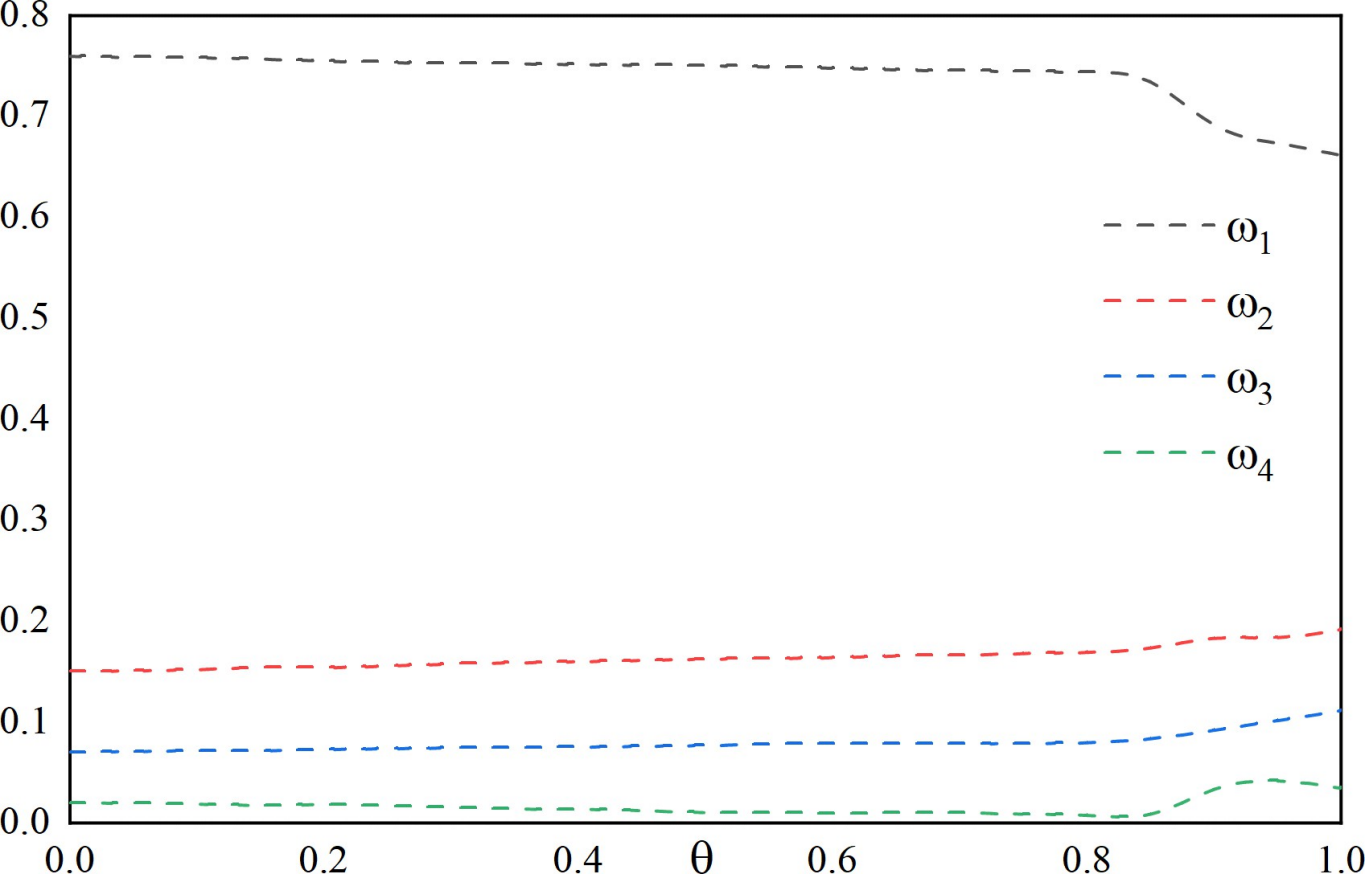
Effect of changes in *θ* on the weighting factor (fixed *β* = 0, *λ* = 1).

**Fig 13:**
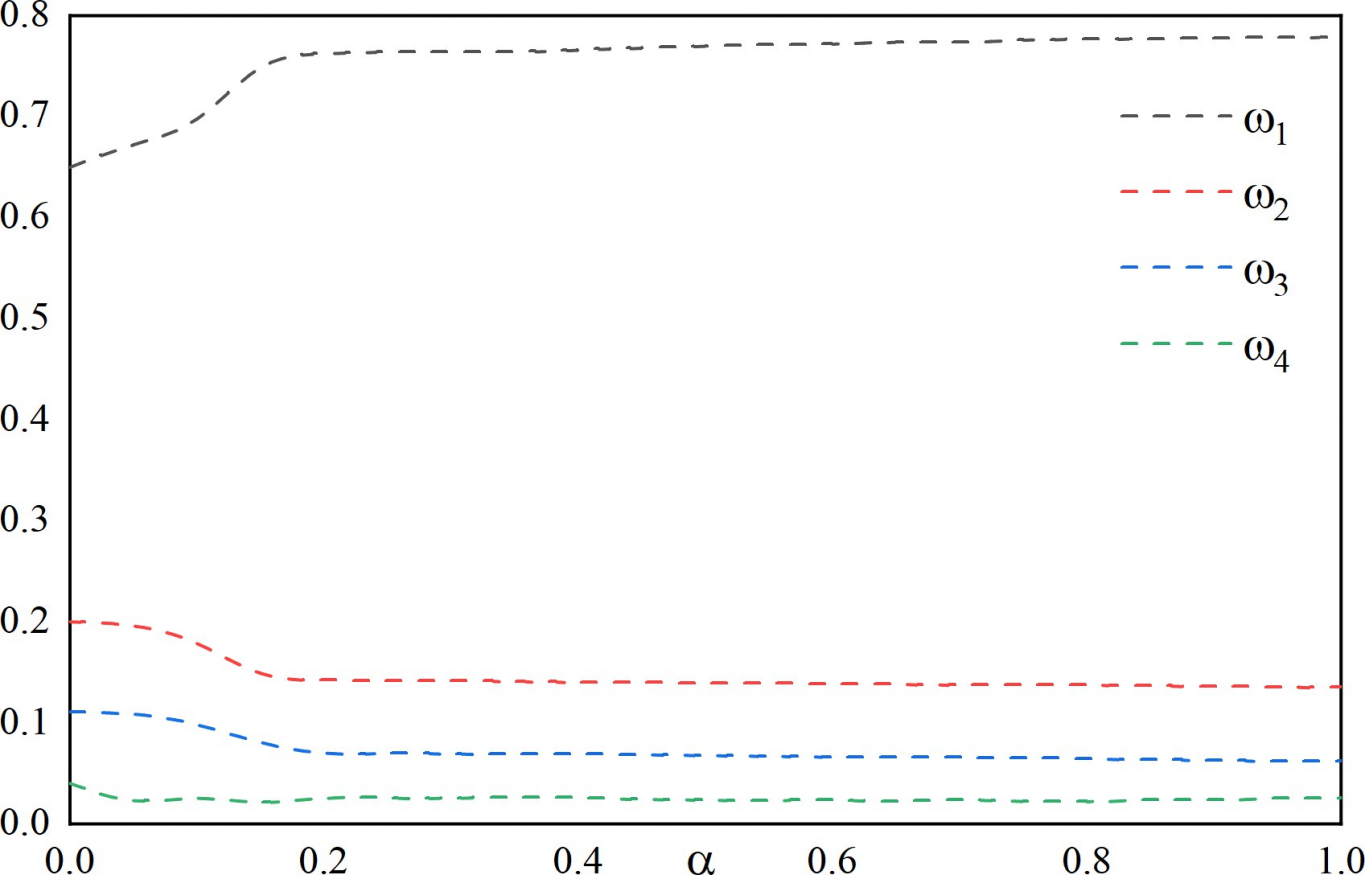
Effect of changes in *α* on the weighting factor (fixed *θ* = 0, *λ* = 1).

In order to observe the effect of variation of preference coefficient *α*, *β*, *θ* between the interval [0,1], first, *α* = 1 is fixed, *β* and *θ* = 1−*β* are varied, the variation as shown in **Fig 11**. Then, *β* = 0 is fixed, *θ* and *α* = 1−*θ* are varied the changes shown in **Fig 12**. Finally, *θ* = 0 is fixed, *α* and *β* = 1−*α* are varied, the changes shown in **Fig 13**.

It can be seen by **Fig 11** that with the increase of the preference coefficient *β*, which takes the value of [0,0.4], *ω*_1_, *ω*_4_ start to decrease and then increase, and *ω*_2_, *ω*_3_ start to increase and then decrease. The *ω*_1_, *ω*_2_, *ω*_3_, *ω*_4_ weight coefficients are relatively stable when taken at (0.4,1].

It can be seen through **Fig 12** that the weight coefficients *ω*_1_,*ω*_2_,*ω*_3_,*ω*_4_ tend to stabilize as the preference coefficient *θ* increases for values taken at [0,0.8], and for values taken at (0.8,1], *ω*_1_ begins to decrease and *ω*_2_,*ω*_3_,*ω*_4_ begins to increase.

It can be seen through **Fig 13** that with the increase of the preference coefficient *α*, taking the value of [0,0.2], *ω*_1_ gradually increases, *ω*_2_, *ω*_3_, *ω*_4_ gradually decreases, and taking the value of (0.2,1], the weight coefficients *ω*_1_, *ω*_2_, *ω*_3_, *ω*_4_ are relatively stable.

By looking at **Fig 11** through **Fig 13**, a general range of values for the preference coefficients is given based on the graphical changes. It can be noticed that when fixing *α*, the value of *β* should be taken as large as possible. When fixing *β*, the value of *θ* is taken as small as possible since starting *θ* from 0 stabilizes the weighting coefficients. When fixing *θ*, in order to satisfy both the values of *α* as well as *β* to ensure the stability of the weight coefficients, the value of *α* should start from 0.2 and should not vary too much. In summary this gives a rough range of values for the coefficients of preference, where *α*∈[0.2,0.4], *β*∈[0.6,0.95], *θ*∈[0,0.05], and *α*+*β*+*θ* = 1.

## 6. Conclusions

The safe operation of coal mines depends on supply of stable air volume, and accurate, reliable forecasts of mine tunnel air volume can significantly enhance the management of coal mine ventilation system. However, the initial tunnel air volume is not stable and can fluctuate due to various factors, making accurate prediction of tunnel air volume a challenging task. To address this challenge, this research proposes an interval combination prediction model for mine tunnel air volume.

In this study, first, the sequence values of the eight influencing factors, along with the collected air volume data are represented interval numbers. This representation eliminates the impact of inconsistent sensor collection and uploading times on air volume prediction. Second, the original nine interval sequence values are decomposed into several intrinsic modal function signals and a residual term using an In-CEEMDAN data preprocessing approach. The Pearson correlation coefficient approach is then used to identify the five influencing factors most strongly correlated with the tunnel’s air volume. Then, the phase space reconstruction is performed on six different types of sequence values using the Wolf method. The Lyapunov values for each type of sequence after reconstruction are then determined, revealing that the values of the six different types of interval sequences are all greater than zero indicating that the chaotic nature of sequences persists even after phase space reconstruction. These sequence values obtained from phase space reconstruction are then input into each of the three single prediction models for air volume prediction.

In particular, the combination prediction framework forms the foundation for the interval combination model for air volume proposed in this research. It considers the peculiarities of the air volume uncertainty in the mine tunnel and transforms the projected interval air volume values into triangular fuzzy number representations. The optimal multi-objective planning model is then constructed using the GIOWLA operator and the gray correlation as the optimality criteria for the triangle fuzzy number of air volume. This multi-objective planning model is then transformed into a single-objective planning problem by imposing limits and introducing preference coefficients. Furthermore, the L2 paradigm is combined with the gray correlation to avoid the issue of prediction error amplification or reduction. This avoids the circumstance in which the air volume interval value’s left endpoint is greater than its right terminus.

The combined model outperforms compared to the traditional prediction model in terms of prediction accuracy and performance. By comparing the predictions made by the three individual prediction methods with the combined prediction, it is evident that the latter significantly improves the accuracy of air volume prediction in underground mine tunnels.
